# Colony-Level Viral Load Influences Collective Foraging in Honey Bees

**DOI:** 10.3389/finsc.2022.894482

**Published:** 2022-05-17

**Authors:** Hannah J. Penn, Michael D. Simone-Finstrom, Lilia I. de Guzman, Philip G. Tokarz, Rachel Dickens

**Affiliations:** ^1^USDA ARS, Sugarcane Research Unit, Houma, LA, United States; ^2^USDA ARS, Honey Bee Breeding, Genetics and Physiology Research Laboratory, Baton Rouge, LA, United States

**Keywords:** *Apis mellifera*, Black queen cell virus (BQCV), Deformed wing virus (DWV), honey bee, macronutrient, nectar, pollen, social immunity

## Abstract

Nutrition is an important component of social insect colony health especially in the face of stressors such as parasitism and viral infections. Honey bees are known to preferentially select nectar and pollen based on macronutrient and phytochemical contents and in response to pathogen loads. However, given that honey bees live in colonies, collective foraging decisions may be impacted directly by forager infection status but also by colony health. This field experiment was conducted to determine if honey bee viral infections are correlated with pollen and nectar foraging and if these associations are impacted more by colony or forager infection. By comparing regressions with and without forager and colony variables and through structural equation models, we were able to determine the relative contributions of colony and forager virus loads on forager decisions. We found that foragers had higher numbers and levels of BQCV and CBPV but lower levels of DWV viruses than their respective colonies. Overall, individuals appeared to forage based a combination of their own and colony health but with greater weight given to colony metrics. Colony parasitism by *Varroa* mites, positively correlated with both forager and colony DWV-B levels, was negatively associated with nectar weight. Further, colony DWV-B levels were negatively associated with individually foraged pollen protein: lipid ratios but positively correlated with nectar weight and sugar content. This study shows that both colony and forager health can simultaneously mediate individual foraging decisions and that the importance of viral infections and parasite levels varies with foraging metrics. Overall, this work highlights the continued need to explore the interactions of disease, nutrition, and genetics in social interactions and structures.

## Introduction

Honey bees [*Apis mellifera* Linnaeus (Hymenoptera: Apidae)] are important pollinators for agricultural production. In North America, particularly in high-value crops such as almonds, their contribution is an estimated $1 billion annually (1, 2). However, in recent years, concerns over colony health have increased based on the rates of annual colony loss (40% in 2017–2018) (3, 4). Nutrition is a critical component of colony health in honey bees [*Apis mellifera* Linnaeus (Hymenoptera: Apidae)] as it impacts the production of brood, overwintering survival, and disease susceptibility (5–10). Pollen and nectar assist in regulating bee immune response, enabling the bee to maintain functionality despite stressors (8, 11). For instance, pollen consumption upregulates vitellogenin expression associated with bee health (12), while honey (processed nectar) consumption upregulates detoxification genes (13).

Within the honey bee colony, foragers are responsible for scouting and collecting food items like nectar and pollen then transporting items back to the nest where items will be distributed or stored by food storing bees for later consumption (14, 15). The need for collective foraging is communicated to foragers through the availability of food storer bees and storage sites as well as chemical cues obtained *via* trophallaxis and olfactory signals (14, 16–20). Olfactory cues from previously obtained food items may also prime foragers to select specific foraging sites or target particular plant species (21, 22).

Once in the field, foragers are able to assess the nutritional quality and quantity of food items, recruiting more foragers to productive patches and plants (23–26). For instance, pollen can vary greatly in macronutrient content (12–60% protein, 1–20% lipid) (27, 28), which can then be detected and selectively foraged by honey bees (29–32). Collectively foraged food items are then returned to the nest, where, after storage, nurse bees feed developing larvae (though they do not necessarily assess macronutrient contents themselves) (33–35).

Food items from different plant taxa have differential impacts on honey bee immune pathways and susceptibility to stressors (5, 36–40). For instance, sunflower pollen has been shown to decrease *Crithidia* replication in bees and alter immunity-related gene expression (41–43). Similarly, a combination of clover and partridge pea pollen reduces virus-induced mortality relative to single source pollen (44). Whereas, bees consuming eucalyptus pollen experienced lower expression of immune genes but higher loads of *Nosema* compared to bees consuming polyfloral pollen (45). Food source interactions with immune pathways may be due, in part, to particular macronutrient profiles that enable bees to overcome stressors (6, 46–48). Protein helps repair and build tissues while lipids provide energy for bees (12, 38). Bees given pollen with lower protein: lipid (P:L) ratios had higher survival under stress than those given pollen with higher P:L ratios (49). Similarly, pollen containing higher fat content was found to reduce Deformed wing virus (DWV) levels (50).

The type of pathogen or parasite, however, appears to impact how immune response interacts with different macronutrients, which is not unexpected as honey bee viruses differ in how they are transmitted, where within the body they proliferate, and associated symptoms (51, 52). Fungal pathogens induced lipid-rich pollen foraging, which increased survival of infected individuals (53). Whereas, pollen containing higher protein levels has been shown to alter bee immune responses to *Nosema* spp. infections, increasing survival and diminishing spore loads (41, 54–56). Similarly, pollen high in protein and amino acids can confer protection from Israeli acute paralysis virus (IAPV)-induced bee mortality (57). Infection or parasitism itself may alter forager food preferences (58). Healthy honey bee foragers exhibit a greater frequency and duration of foraging trips and focus more on pollen than nectar relative to foragers infected with *Nosema apis* (Zander) (37).

Foraging for plant-derived resources may be influenced by infection at the colony or individual bee level (9, 59–62). Bee foragers face the dilemma on whose needs to forage for—themselves or the colony as a whole—as foragers are responsible for communicating information about floral resources to the rest of the colony in addition to their collective foraging tasks (63–65). In ants, foragers selecting high-carbohydrate diets can increase levels of social immunity against fungal pathogens in the colony with individual foragers benefitting from group immunity and colony-level nutrition (66). Honey bee resin foraging has been noted to be influenced by colony-level *Varroa* mite infestation (61), providing suggestive evidence that colony infection/infestation status influences individual foraging (59). Despite clear colony drivers in some foraging decisions, individual infection status (in terms of preference or ability) could override these priorities (47, 67), meaning that forager needs may not be entirely aligned with colony-level benefits (68). For instance, *Nosema*-infected honey bees have been shown to forage for items that enhance their own health status (69); and the same may hold true for honey bees facing viral challenges (70, 71).

Given that colony health relies both on collective foraging and social immunity, but these facets are mediated by individual bees, we wanted to assess the interplay of these two aspects of colony life. First, we determined if honey bees forage on macronutrients based on infection status; and then we assessed if foragers altered their preferences in response to their own or colony viral loads. This was overlayed with a preliminary investigation of variation in these responses among different honey bee stocks (i.e., distinct commercially produced populations or specific lines bred for particular traits of interest such as resistance to *Varroa* mite parasitism). To do this, we evaluated individual pollen foraging in terms of protein and lipid content, individual nectar foraging (quantity and sugar content), as well as colony and forager viral loads. We predicted that increased mite and viral loads would result in an increase in collected pollen protein content (41, 72). Further, given that viral infections also pre-dispose Hymenopterans to increased foraging on high-sugar sources (37, 73), we expected that higher virus levels would result in higher sugar content of nectar loads. Prior work has indicated that individual action may be based on a combination of individual ability (67, 69) and social immunity or collective foraging requirements (9, 59–62). But given that individual health status influences how well an individual can forage for the colony, we expected that foragers would collect food items based primarily individual rather than colony infection status.

## Materials and Methods

### Source Colonies and Sample Collection

An apiary was established at the Baton Rouge, LA, USDA Honey Bee Breeding, Genetics and Physiology Research Laboratory (30°22′56″N, 91°10′40″W) with nine colonies (three from each of the following stocks: Italian, Pol-Line, and Russian) using packaged bees (~0.90 kg or 7,000 worker bees per package). Pol-Line and Russian queens were sourced from the same USDA lab; mite-susceptible Italian queens were sourced from a commercial queen producer. Packages were installed on 23 April 2019 and supplied with sugar water for the first month *via* in-hive feeders. Colonies were equalized as necessary on 13 May and 5 June with additional brood and food (combination of pollen and nectar) frames. Colonies were sampled a minimum 6 weeks after the last brood supplement to allow time for the worker populations to reflect queen genetics. Colony and pollen forager sampling was completed once per month during the weeks of 22 July (time 1), 19 August (time 2), and 9 September 2019 (time 3). Nectar foragers were only collected for time 1 to streamline later collection periods.

Colony-level pollen preferences were determined using pollen traps for 4 days, with the pollen collected daily, mixed with previous days' samples from the same colony for that sampling period, and stored at −20°C for nutritional analyses. Throughout the course of study observation, colonies were not treated with miticide in order to obtain an accurate representation of naturally occurring *Varroa* mite levels. To estimate *Varroa* mite levels per 100 bees, ~300 nurse bees were sampled from brood frames per colony (stored at −20°C), washed using detergent, then bees and mites were counted (74, 75). A pool of 50 adult bees per colony was simultaneously sampled from two brood frames then stored at −80°C for colony viral analyses.

To sample nectar and pollen foragers, colony entrances were closed between 09:00 and 11:00, and returning foragers were collected using scintillation vials (1 forager/vial). Each vial was placed on ice for 2 min to reduce movement and prevent swallowing of the honey stomach contents. Pollen loads were removed from the corbiculae of each bee bearing pollen (*N* = 24/colony/time) and stored at −20°C for nutritional analyses. For each nectar forager (*N* = 24/colony), honey stomach content was collected into a pre-weighed microcapillary tube by gently squeezing the abdomen. Nectar load was assessed *via* weight measurements of the microcapillary tubes after sample collection (76). Microcapillary tube contents were then exuded onto a digital refractometer (MISCO Palm Abbe Digital Fluid Refractometer, Solon, OH, USA) for analysis of sugar content (Brix) (77). Due to maximum refractometer detection limitations, nectar loads >85% sugar content were designated as >85%. All collected pollen and nectar foragers were stored individually at −80°C for subsequent forager viral analysis.

### Pollen Nutritional Analyses

For colony-level pollen nutritional analyses, 10 g of pollen collected *via* pollen traps from each colony for each time point were sent to the Agricultural Chemistry Laboratory (Louisiana State University, AgCenter, Baton Rouge, LA, USA) for analyses of crude protein (5 g) and fat content (5 g). For forager-level pollen analyses, pollen pellets (from both corbiculae of the same individual bee) were weighed and then one pellet was analyzed for protein content and one for lipid content. For protein analysis, pellets were individually homogenized using a handheld pestle, vortexed, and analyzed for total soluble protein using a Bradford Assay (Bio-Rad DC protein assay kit). Protein standards (0.28, 0.24, 0.2, 0.16, 0.12, 0.08, 0.04, and 0 mg/mL) were created using BSA (Bio-Rad). Five microliters of each sample and standard in triplicate were combined with 245 μL of Bradford reagent (Bio-Rad), incubated for 5 min, and analyzed using a spectrophotometer (SpectraMax Plus Microplate Reader) at 595 λ. For analysis of lipid content, pellets were individually dried in a desiccator at room temperature for 24 h, weighed, and analyzed using a modified chloroform-extraction (78, 79). Samples were vortexed with 0.2 mL 2% sodium sulfate. One milliliter of chloroform-methanol (2:1) was added, vortexed, then centrifuged 2,180 G for 5 min at room temperature. Three hundred microliters of the layer below the supernatant was combined with 0.6 mL of deionized water, vortexed, centrifuged again, and incubated at 90°C for 20 min. Three hundred microliters of 10 N sulfuric acid (Thermo Fisher Scientific, Waltham, MA, USA) was added, then samples were incubated at 90°C for 20 min followed by a 2-min ice bath for samples to reach room temperature. Hundred microliters of each sample was read on a spectrophotometer (SpectraMax Plus Microplate Reader) at 540 λ. Samples were compared against canola oil standards (0.20, 0.18, 0.15, 0.12, 0.09, 0.06, 0.03, and 0.015 mg/mL).

### Viral Analyses

For colony-level viral analyses, nurse bee pools (*N* = 27 samples each, representing a pool of 50 bees/colony to generally approximate colony viral loads) were placed in 50 mL homogenization vials pre-fitted with ceramic beads then homogenized using a bead mill (Omni-Inc Bead Ruptor Elite, Kennesaw, GA, USA). For forager-level viral analyses, a subset of bees (*N* = 378 foragers total) from each colony and time was selected based on being at the high (*N* = 162 bees, *N* = 6 bees/colony/time) and low (*N* = 162 bees, *N* = 6 individual bees/colony/time) range of individually collected pollen protein levels or the high (*N* = 27 bees, *N* = 3 individual bees/colony) and low (*N* = 27 bees, *N* = 3 individual bees/colony) range of sugar content (Brix). Individual bees were homogenized by hand at 5°C in a metal bead bucket using sterilized plastic pestles. Following homogenization, 200 μL of Promega Lysis buffer and 200 μL of Promega Homogenization solution (Promega Corporation, Madison, Wisconsin, USA) were added to each sample. All samples were briefly vortexed then centrifuged for 10 min at 4°C at 14,000 rpm. Total sample RNA was then extracted from 400 μL cleared lysate using the Maxwell RSC 48 simplyRNA cartridges per manufacturer recommended protocol (Promega Corporation, Madison, Wisconsin, USA). RNA was stored in 0.6 mL elution tubes wrapped in parafilm (Bemis NA, Neenah, Wisconsin, USA) at −80°C until cDNA synthesis.

Before cDNA synthesis, frozen RNA samples were thawed on 5°C metal beads, briefly vortexed, then centrifuged. Each RNA sample was quantified *via* spectrophotometry (NanoDrop One, Thermo-Fisher Scientific Inc., Waltham, Massachusetts, USA) twice using 1 μL of sample each time. The mean ng/μL NanoDrop One readings were calculated per sample then used to determine the volume of sample RNA template and nuclease-free water required to reach a sample concentration of 100 ng of RNA. cDNA was then synthesized using 0.2 mL PCR strip tubes in two steps using Qiagen Quantitect Reverse Transcriptase kits (Thermo-Fisher Scientific Inc., Waltham, Massachusetts, USA). For step one, 2 μL of gDNA wipeout was added to the mix of RNA and water for a total reaction volume of 14 μL per sample. Samples were incubated per manufacturer protocol at 42°C for 2 min in a Bio-Rad T100 Thermal Cycler (Bio-Rad, Hercules, California, USA). Samples were then briefly vortexed and centrifuged before the addition of 6 μL of Step 2 Master Mix consisting of 4 μL 5X Buffer, 1 μL of RT Primer mix, and 1 μL of RT enzyme per sample. Samples were again briefly vortexed and centrifuged then placed into the Bio-Rad T100 Thermal Cycler (42°C for 25 min then 95°C for 3 min) per manufacturer protocol. Transcribed cDNA was in strips tubes were wrapped in parafilm and stored at −80°C until RT-qPCR.

For analyses of both colony and forager viral profiles, samples were analyzed for eight viruses (primers in Table 1): Acute bee paralysis virus (ABPV), Black queen cell virus (BQCV), Chronic bee paralysis virus (CBPV), Deformed wing virus genotype A (DWV-A), Deformed wing virus genotype B (DWV-B), Israeli acute paralysis virus (IAPV), Kashmir bee virus (KBV), and Lake Sinai virus (LSV), following well-established protocols (85–87). The reference gene β-actin was used to ensure sample quality (88). Each sample was replicated three times per primer pair for RT-qPCR analyses. All RT-qPCR reactions consisted of 5 μL SsoFast Universal SYBR Green supermix (Bio-Rad, Hercules, California, USA), 3 μL nuclease-free water (Promega Corp., Madison, WI), 0.5 μL forward primer, 0.5 μL reverse primer, and 1 μL cDNA from each sample. All reactions were run in Bio-Rad CFX96 or CFX Connect Thermal Cyclers (Bio-Rad, Hercules, California, USA) with all reactions of a specific primer occurring in the same machine. The PCR thermal cycling protocol for the DWV-A and CBPV primer pairs was 95°C for 5 min followed by 40 cycles of 95°C for 5 s and 53.5°C for 10 s then 72°C for 10 s; while the protocol for ABPV, β-actin, BQCV, DWV-B, KBV, and LSV was 95°C for 5 min followed by 40 cycles of 95°C for 5 s and 52.5°C for 10 s then 72°C for 10 s. The PCR cycling protocol for the IAPV primer pairs was 95°C for 5 min followed by 40 cycles of 95°C for 5 s and 53.5°C for 10 s then 72°C for 10 s. The thermal protocols included a melt-curve dissociation analysis to confirm product size. BQCV, CBPV, DWV-A, and DWV-B results were quantified using the Standard Curve Method using linearized plasmid constructs for pooled colony samples as well as individual nectar and pollen foragers. Quantified virus levels were log transformed for analyses and graphical representation; all other viruses were counted as positive for any cycle threshold (Ct) value registered at <40 cycles.

**Table 1:** Primers used for virus detection and sample quality.

**Primer target**	**Forward sequence**	**Reverse sequence**	**References**
ABPV	CTTTCATGATGTGGAAACTCC	AAACTGAATAATACTGTGCGTA	Francis and Kryger (80)
BQCV	TTTAGAGCGAATTCGGAAACA	GGCGTACCGATAAAGATGGA	Boncristiani et al. (81)
CBPV	CGCAAGTACGCCTTGATAAAGAAC	ACTACTAGAAACTCGTCGCTTCG	Blanchard et al. (82)
DWV-A	GAGATTGAAGCGCATGAACA	TGAATTCAGTGTCGCCCATA	Boncristiani et al. (81)
DWV-B	CTGTAGTTAAGCGGTTATTAGAA	GGTGCTTCTGGAACAGCGGAA	Ryabov et al. (83)
IAPV	GCGGAGATTATAAGGCTCAG	CCTGCAAGATAAGAAAGGGGG	Francis and Kryger (80)
KBV	TGAACGTCGACCTATTGAAAAA	TCGATTTTCCATCAAATGAGC	Francis and Kryger (80)
LSV	CGTGCGGACCTCATTTCTTCATGT	CTGCGAAGCACTAAAGCGTT	Daughenbaugh et al. (84)
β-actin	AGGAATGGAAGCTTGCGGTA	AATTTTCATGGTGGATGGTGC	Ryabov et al. (83)

### Statistical Analyses

All analyses were conducted in JMP Pro 16.0.0 Pro (SAS Institute, Cary, NC, USA). Forager viral loads were compared to those of their respective colonies with the Kruskal-Wallis test. General associations among variables were determined using Spearman's rank correlation coefficient. To determine contribution of individual and colony level virus loads to individual foraging (% protein, % lipid, protein to lipid ratio (P:L), and nectar weight], we used the generalized regression function with a gaussian distribution. Independent variables included sampling month, *Varroa* mites per 100 bees (mites), and virus panel data [the total number of viruses (Virus), BQCV levels, CBPV levels, DWV-A levels, and DWV-B levels] for both the foragers and the colony as a whole. The variables representing the presence/absence of ABPV, BQCV, CBPV, IAPV, LSV, and KBV were not included as variation was not large enough to be meaningful, but these data can be found in the Supplementary Material. Due to the 85% cutoff for Brix measurements, nectar sugar content was grouped into low (0–33.0%), medium (33.1–66.0%), and high (66.1–85%) categories. The contribution of virus panel data to sugar content was evaluated using an ordered logit model in the generalized regression function using the same independent variables as above. Bee stock was added to some figures for demonstration purposes but was not included in the models due to small sample size per stock.

The relative contribution of forager and colony disease load to individual forager selection was initially evaluated by using model selection criteria (-log likelihood, AICc, BIC, and *R*^2^). The full model (as described above) was compared against models including only forager and only colony virus panel data. The relative contribution of colony vs. individual forager viral infections to individual foraging decisions was further analyzed using structural equation models (SEMs). In SEMs, measured variables (manifest variables) can be combined into a larger representative latent variable that describes some larger, unmeasured factor (89–91). Based on our *a priori* hypothesis that individual and colony level disease can contribute differentially to foraging preferences, we contrasted two sets of models for nectar and pollen foraging. Individual forager (i) and colony (c) manifest variables (virus number and log-transformed BQCV, CBPV, DWV-A, and DWV-B levels) were combined into two respective latent factors representing viral infection of the two sources (Forager and Colony). To account for potential interactions between observed colony and individual viral infection, all models allowed Forager and Colony latent factors to covary without specifying directionality. For nectar foragers, we modeled both Forager and Colony disease latent factors with direct relationships to a Nectar latent factor (nectar weight and sugar content manifest variables). We also repeated the nectar SEMs with nectar weight and sugar content manifest variables individually. As these models were not a good fit for nectar, we only present them in the Supplementary Materials. For pollen foragers, we modeled time as directly related to both latent disease factors and modeled both latent disease factors with direct relationships to the Pollen latent factor (% protein and % lipid manifest variables). We then modeled the latent disease factors against the individual pollen manifest variables. Model fit was assessed with a combination of the comparative fit index (CFI), root mean square error of approximation (RMSEA) with priority given to models closest to optimal values of CFI (>0.9) and RMSEA (<0.1) (92, 93). Models were estimated using maximum likelihood with 1,000 iterations. All SEM results are presented as standardized estimates to directly compare the contributions of colony and individual forager viral infection to foraging decisions.

## Results

### Viral Loads and *Varroa* Infestation Rate

#### Colony Varroa and Viral Loads

Colony mite levels were generally very low (mean 3.218 ± 1.004 mites/100 bees), with an increase over time (July: 1.271 ± 0.342; August: 2.281 ± 0.635; September: 6.102 ± 2.772). The higher mite levels in September were driven primarily by two colonies with mites loads over 20 mites per 100 bees (one Italian and one Russian colony). Colonies were infected with a mean of 5.123 ± 0.152 viruses over the entire observation period (Supplementary Tables 1, 2). Colony virus number was positively correlated (using Spearman's ρ) with colony levels of CBPV (Table 2). Colony DWV-B levels were also positively correlated with colony CBPV levels and *Varroa* mite levels (Table 2).

**Table 2:** Spearman's ρ correlation values for colony (c) and individual pollen forager (i) values of pollen protein, lipid, P:L ratio, virus number, and viral loads of BQCV, CBPV, DWV-A, and DWV-B.

	**c_Prot**	**c_Lipid**	**c_PL**	**c_mite**	**c_VirusNo**	**c_BQCV**	**c_CBPV**	**c_DWV-A**
c_Lipid	−0.084							
c_PL	0.330	−0.933*						
c_mite	−0.273	−0.339	0.246					
c_VirusNo	0.137	−0.337	0.165	0.154				
c_BQCV	0.154	−0.308	0.318	0.133	0.078			
c_CBPV	−0.053	−0.059	−0.023	0.183	0.395*	−0.112		
c_DWV-A	0.113	0.349	−0.319	−0.280	−0.295	0.040	0.054	
c_DWV-B	−0.195	−0.101	−0.049	0.403*	0.156	0.323	0.482*	−0.091

*Statistical significance (P < 0.05) is indicated by asterisks*.

#### Pollen Forager Viral Loads

**Figure 1:**
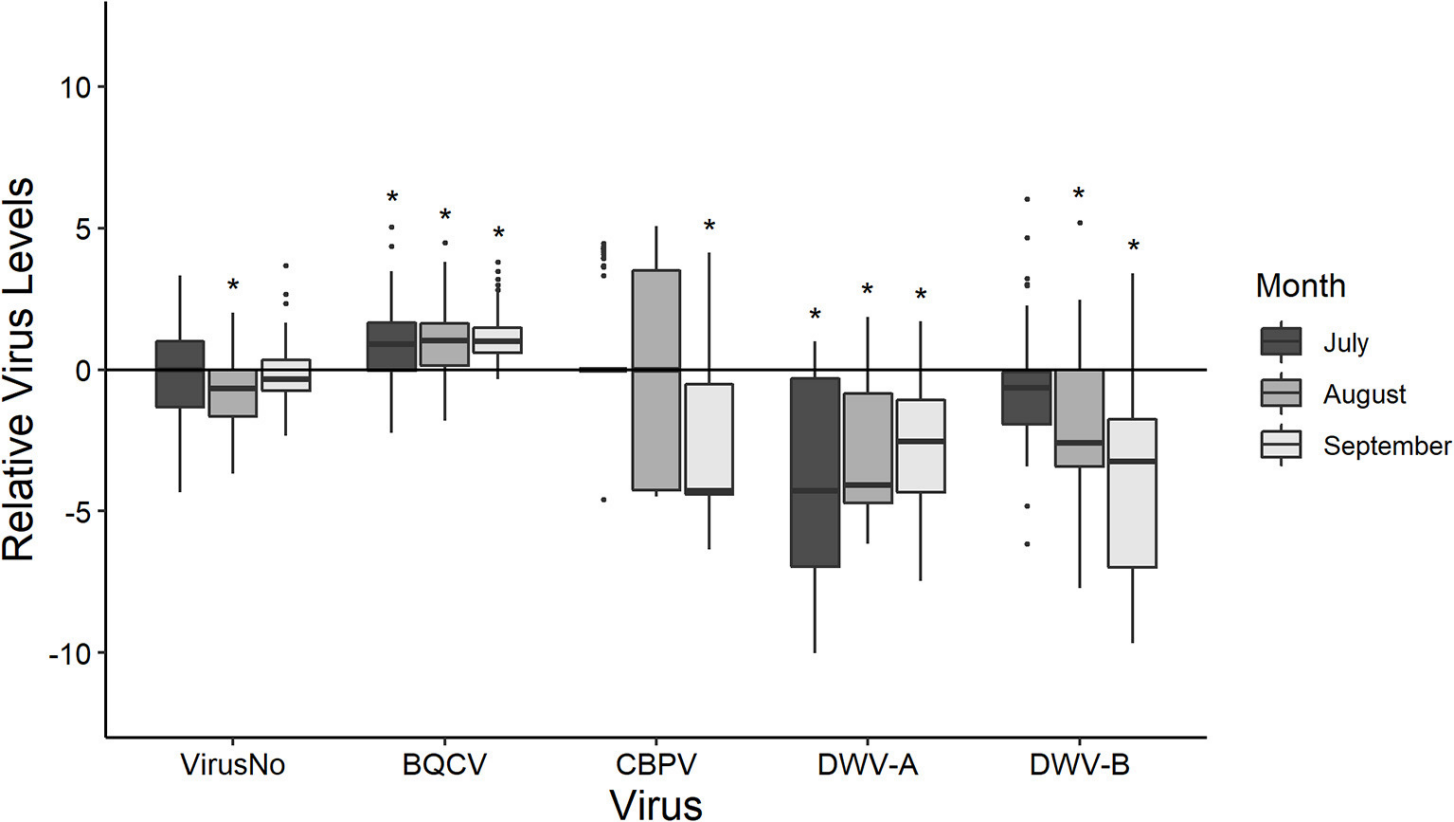
The disease levels of pollen foragers scaled to that their respective colonies at each time point (y-axis = 0). Boxplots are in the style of Tukey, with horizontal lines indicating median values while box limits indicate upper and lower quartiles. This was conducted for the total number of viruses (VirusNo) detected within the sample, log-transformed BQCV, CBPV, DWV-A, and DWV-B levels. Asterisks indicate significant differences between forager values and colony values as determined by Kruskal-Wallis test (*P* < 0.05).

Pollen foragers often differed in their virus loads from the pooled colony samples with the exception being total virus number (Figure 1). Pollen foragers exhibited an overall mean of 4.706 ± 0.027 viruses (Supplementary Tables 3, 4). When split out by observation month, pollen foragers did not differ from colonies in virus number except in August (July: *P* = 0.475; August: *P* = 0.020; September: *P* = 0.650). Overall, pollen foragers (10^5.647±0.021^) had greater BQCV than the colony samples (10^4.713±0.181^) with all timepoints being different (July: *P* = 0.015; August: *P* = 0.004; September: *P* < 0.001). CBPV was similar in colonies (10^1.895±0.455^) and pollen foragers (10^0.751±0.039^) over time except for September (July: *P* = 0.972; August: *P* = 0.536; September: *P* < 0.001). Foragers had lower levels of DWV-A (10^4.217±0.026^) and DWV-B (10^6.581±0.055^) than their associated colonies (DWV-A: 10^7.555±0.486^; DWV-B: 10^8.880±0.449^). This held for all time points (DWV-A: July: *P* < 0.001; August: *P* < 0.001; September: *P* < 0.001; DWV-B July: *P* = 0.149; August: *P* = 0.009; September: *P* < 0.001).

Pollen forager virus loads were associated (using Spearman's ρ) with other viral infections at the individual level as well as those at the colony level (Table 3). Individual forager virus number was positively correlated with individual DWV-A and DWV-B levels as well as colony CBPV levels. Forager BQCV was negatively correlated with forager DWV-B levels, colony mite levels, and colony CBPV levels. Forager BQCV was only positively correlated with colony BQCV levels. Similarly, forager CBPV positively correlated with colony CBPV levels. Forager CBPV levels were negatively correlated with forager DWV-A and DWV-B levels as well as colony CBPV. Forager DWV-A was positively associated with individual DWV-B, colony CBPV, and colony DWV-B but was not associated with colony DWV-A. Forager DWV-B levels were positively correlated with all colony metrics except for colony BQCV, which was a negative association.

**Table 3:** Spearman's ρ correlation values for colony (c) and individual pollen forager (i) values of pollen protein, lipid, P:L ratio, virus number, and viral loads of BQCV, CBPV, DWV-A, and DWV-B.

	**i_Prot**	**i_Lipid**	**i_PL**	**i_VirusNo**	**i_BQCV**	**i_CBPV**	**i_DWV-A**	**i_DWV-B**
i_Lipid	0.461*							
i_PL	0.323*	−0.622*						
i_Virus	0.239*	0.107	0.080					
i_BQCV	−0.194*	0.089	−0.175*	−0.008				
i_CBPV	0.097	0.237*	−0.110	0.313	0.108			
i_DWV-A	0.200*	−0.025	0.219*	0.317*	−0.023	−0.110*		
i_DWV-B	0.095	−0.181*	0.133	0.346*	−0.180*	−0.218*	0.417*	
c_mite	0.105*	−0.066	0.137*	0.044	−0.334*	−0.095	0.027	0.276*
c_VirusNo	0.095*	−0.158*	0.296*	0.084	−0.187*	−0.049	−0.025	0.322*
c_BQCV	−0.227*	−0.169*	0.100	−0.035	0.183*	0.110*	−0.095	−0.372*
c_CBPV	0.309*	−0.024	0.243*	0.161*	−0.283*	−0.203*	0.324*	0.377*
c_DWV-A	0.024	0.036	−0.057	−0.019	0.071	−0.050	0.022	0.118*
c_DWV-B	0.393*	0.249*	0.095	0.075	−0.252*	−0.062	0.239*	0.220*

*Statistical significance (P < 0.05) is indicated by asterisks*.

#### Nectar Forager Viral Loads

**Figure 2:**
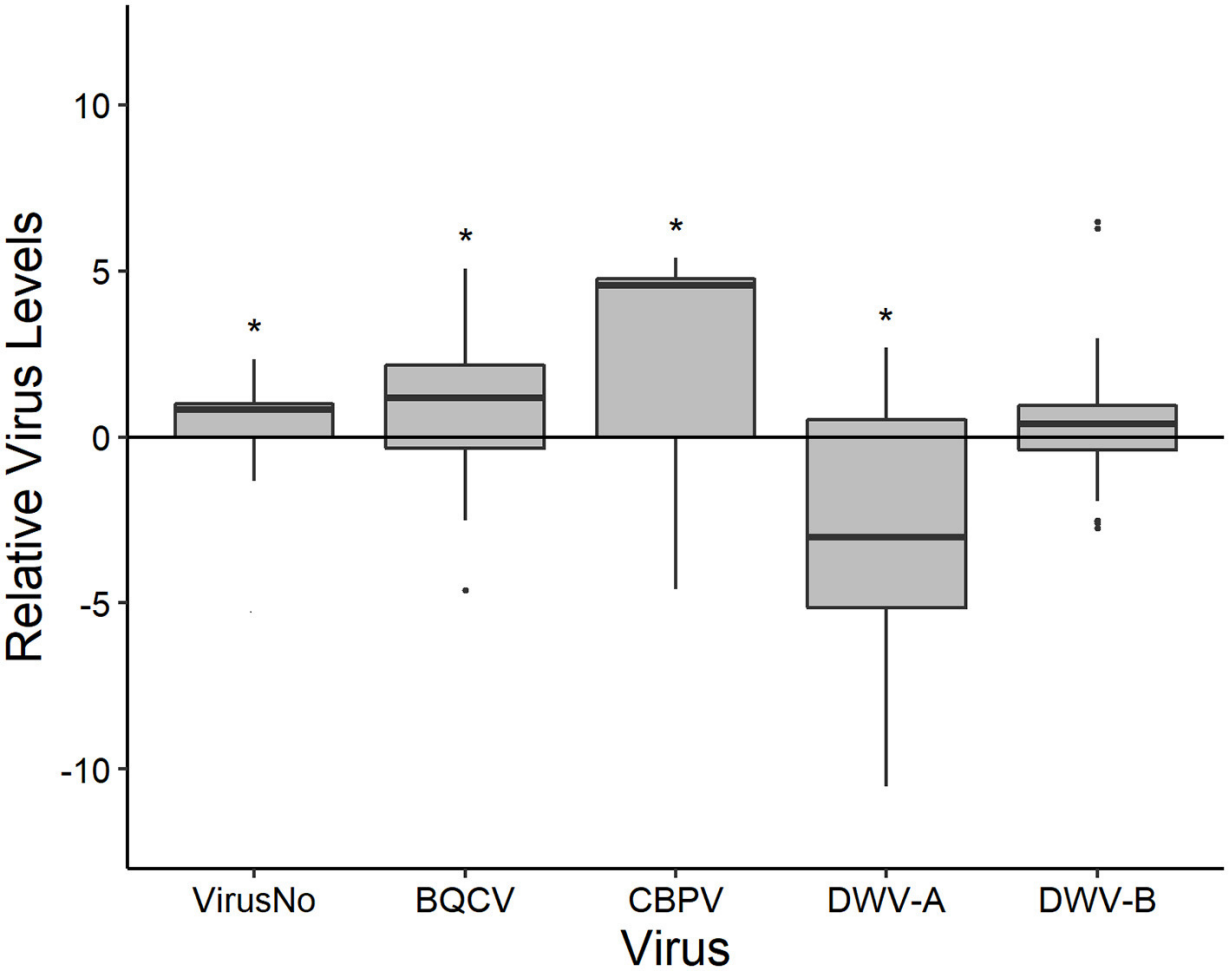
The disease levels of nectar foragers scaled to that their respective colonies (y-axis = 0). Boxplots are in the style of Tukey, with horizontal lines indicating median values while box limits indicate upper and lower quartiles. This was conducted for the total number of viruses (VirusNo) detected within the sample, log-transformed BQCV, CBPV, DWV-A, and DWV-B levels. Asterisks indicate significant differences between forager values and colony values as determined by Kruskal-Wallis test (*P* < 0.05).

Nectar foragers also differed in their virus loads from the pooled colony samples with the exception being DWV-B (Figure 2). Overall, nectar foragers had a greater number of viruses (5.315 ± 0.098; Supplementary Tables 5, 6) as well as levels of BQCV (10^6.185±0.197^) and CBPV (10^2.874±0.349^) than the colony samples. Nectar foragers had lower DWV-A levels (10^5.413±0.129^) but not DWV-B levels (10^7.352±0.231^) compared to the associated colonies.

Nectar forager virus loads were primarily associated (using Spearman's ρ) with viral infections at the individual level (Table 4). Individual nectar forager virus number was positively correlated with forager BQCV, CBPV, and DWV-A levels. Nectar Forager BQCV levels were positively correlated with individual CBPV, and DWV-A while forager DWV-A was positively correlated with forager DWV-B and colony CBPV. Finally, nectar forager DWV-B levels were positively correlated with colony *Varroa* mite and colony DWV-B levels.

**Table 4:** Spearman's ρ correlation values for colony (c) and individual nectar forager (i) values of nectar weight (mg), sugar content (Brix), Virus number, and viral loads of BQCV, CBPV, DWV-A, and DWV-B.

	**Weight**	**Sugar**	**i_VirusNo**	**i_BQCV**	**i_CBPV**	**i_DWV-A**	**i_DWV-B**
Sugar	−0.392*						
i_VirusNo	0.085	−0.010					
i_BQCV	0.051	−0.125	0.353*				
i_CBPV	0.023	−0.061	0.752*	0.331*			
i_DWV-A	−0.034	−0.161	0.281*	0.272*	0.250		
i_DWV-B	−0.207	−0.059	0.136	0.207	0.067	0.473*	
c_mite	−0.036	−0.153	−0.154	0.106	−0.109	−0.006	0.396*
c_VirusNo	0.007	−0.049	0.084	0.031	0.130	0.172	0.266
c_BQCV	0.426*	−0.231	0.100	−0.001	−0.009	0.269*	0.050
c_CBPV	−0.087	0.018	0.014	−0.034	0.041	−0.034	0.234
c_DWV-A	−0.042	−0.050	−0.150	−0.157	−0.186	−0.157	−0.043
c_DWV-B	0.100	−0.252	0.043	−0.155	−0.061	0.219	0.495*

*Statistical significance (P < 0.05) is indicated by asterisks*.

### Viral Interactions With Foraging

#### Pollen Foraging

##### Protein Content

The range of % protein in pollen dry weight varied more for foragers (0.531–33.948%) than the combined colony sample (10.5–18.4%). The mean protein content of individual pollen pellets (8.789 ± 0.180%) was lower than the pooled colony sample pellets (15.078 ± 0.390%). The protein content of the pollen pellets increased throughout the observation period, with September having significantly greater protein content than August and August having higher levels than July (Tukey HSD, *P* < 0.05).

Protein content of the forager's pollen pellet was correlated (using Spearman's ρ) with a combination of forager and colony virus metrics (Table 3). Forager and colony BQCV statuses were negatively associated with individually foraged pollen protein content. Forager pollen protein content was positively correlated with forager virus number and forager DWV-A. Forager pollen protein content was further correlated with colony *Varroa* mite levels, virus number, CBPV levels, and DWV-B levels. However, when we analyzed protein content of forager-collected pollen using regression, month of collection was the only significant variable (Table 5; Supplementary Table 7).

**Table 5:** Individual pollen forager generalized regression model effect test summaries for the percentage of protein and lipid in the collected pollen (dry weight) as well as the protein to lipid ratio (P:L).

		**Protein**				**Lipid**				**P:L**			
**Type**	**Variable**	**DF**	**Sum squares**	***F***	***P***	**DF**	**Sum squares**	***F***	***P***	**DF**	**Sum squares**	***F***	***P***
Time	Month	2	1,210.612	15.142	0.000	1	53.359	4.178	0.043	1	106.185	5.069	0.026
Forager	Virus no.	1	57.791	1.446	0.230	1	17.862	1.399	0.239	1	2.308	0.110	0.740
	BQCV	1	25.169	0.630	0.428	1	13.585	1.064	0.304	1	0.128	0.006	0.938
	CBPV	1	123.599	3.092	0.080	1	39.417	3.086	0.081	1	14.400	0.687	0.409
	DWV-a	1	56.167	1.405	0.237	1	8.547	0.669	0.415	1	4.555	0.217	0.642
	DWV-b	1	32.151	0.804	0.371	1	2.374	0.186	0.667	1	3.107	0.148	0.701
Colony	Mites	1	39.475	0.987	0.321	1	21.854	1.711	0.193	1	19.986	0.954	0.330
	Virus no.	1	0.141	0.004	0.953	1	45.458	3.559	0.061	1	0.403	0.019	0.890
	BQCV	1	7.289	0.182	0.670	1	0.259	0.020	0.887	1	23.871	1.140	0.288
	CBPV	1	22.573	0.565	0.453	1	0.155	0.012	0.912	1	43.297	2.067	0.153
	DWV-A	1	0.719	0.018	0.893	1	0.313	0.024	0.876	1	67.422	3.219	0.075
	DWV-B	1	0.576	0.014	0.905	1	0.439	0.034	0.853	1	101.153	4.829	0.030

##### Lipid Content

The range of % lipid in pollen dry weight varied more for foragers (0.095–15.384%) than the combined colony sample (0.70–3.14%). The mean lipid content of individual pollen pellets (4.966 ± 0.137%) was higher than the pooled colony sample pellets (1.908 ± 0.137%). The lipid content of the pollen increased throughout the observation period, with September having significantly greater lipid content than July (Tukey HSD, *P* < 0.05).

Lipid content of the forager's pollen pellet was also correlated (using Spearman's ρ) with a combination of forager and colony virus metrics, though the directionally if these associations differed based on if they were due to forager or colony infection (Table 3). For instance, while colony DWV-B was positively associated with individually foraged pollen lipid content, forager DWV-B levels were negatively associated with lipid content. Forager CBPV was also positively correlated while colony virus number and colony BQCV were negatively correlated. Again, when we analyzed lipid content of forager-collected pollen using regression, month of collection was the only significant variable (Table 5; Supplementary Table 7), though colony virus number was marginally negatively associated with lipid content (*P* = 0.059), matching the Spearman's ρ.

##### P:L Ratio

The range of individually foraged pollen P:L ratios again varied more for foragers (0.100–30.358) than the combined colony samples (4.342–15.586), potentially as a function of the high variability of both protein and lipid contents in the individually foraged pellets. The mean P:L individual pollen pellets (3.464 ± 0.174) was lower than the pooled colony sample pellets (9.369 ± 0.754). The P:L ratio of the pollen increased throughout the observation period, with September having significantly greater protein content than July (Tukey HSD, *P* < 0.05).

**Figure 3:**
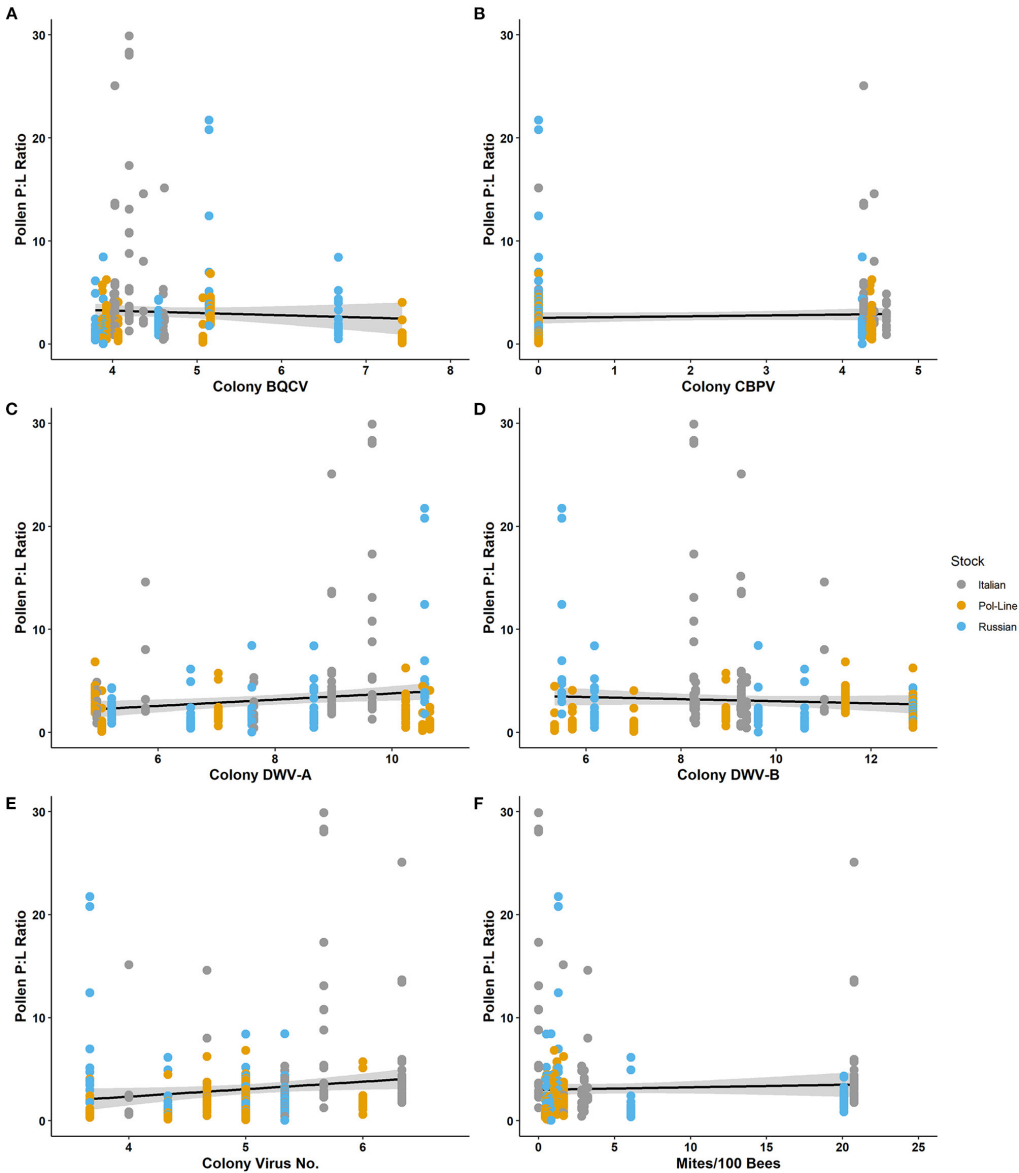
Protein: lipid ratios of individually foraged pollen relative to log-transformed colony level levels of **(A)** BQCV, **(B)** CBPV, **(C)** DWV-A, **(D)** DWV-B, **(E)** virus number, and **(F)** mites per 100 bees.

Like both the individual protein and lipid components, the P:L ratio of the individually foraged pollen pellet was associated (using Spearman's ρ) with a combination of forager and colony virus metrics (Table 3). Individually foraged pollen P:L was positively correlated with forager DWV-A as well as colony virus number and colony CBPV. Further, P:L was negatively associated with forager BQCV. Further, the regression model for P:L content indicated that month of collection and colony DWV-B levels were significant (Table 5; Supplementary Table 7), with higher colony DWV-B levels negatively associated with P:L ratios of individually foraged pollen (Figure 3; Supplementary Figure 1). Some of the P:L ratio interactions with forager and colony viral infections appeared to be contingent on bee stock (Supplementary Figures 5, 6), as aspect requiring further study.

#### Nectar Foraging

##### Nectar Load Weight

**Figure 4:**
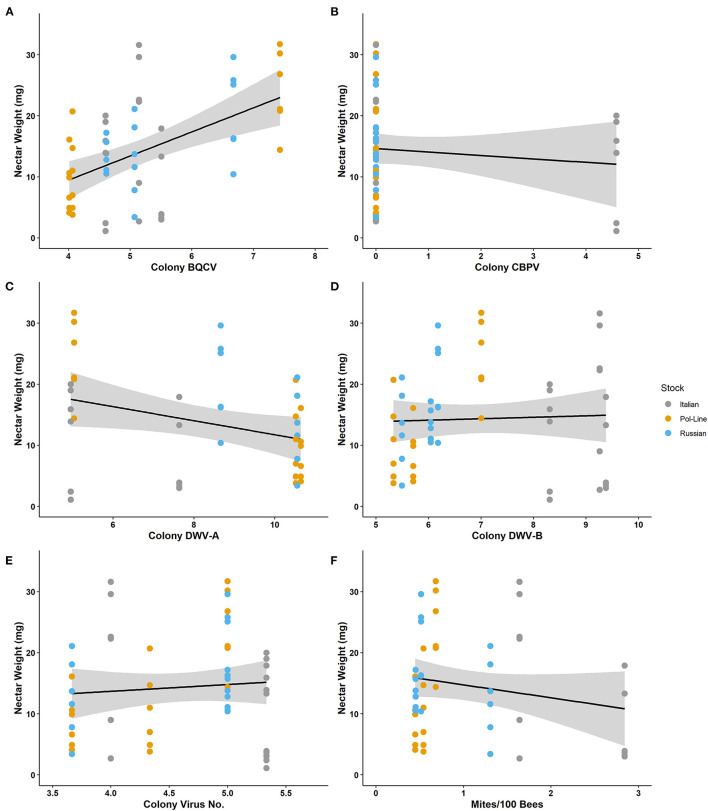
Nectar weight (mg) collected by individual foragers relative to log-transformed colony level levels of **(A)** BQCV, **(B)** CBPV, **(C)** DWV-A, **(D)** DWV-B, **(E)** virus number, and **(F)** mites per 100 bees.

Nectar weight ranged from 1.10 to 31.70 mg with a mean of 14.34 ± 1.16. Nectar weight was positively associated with colony BQCV (Table 4). The regression model for nectar weight also indicated that colony BQCV levels as well as colony mite levels were significant (Table 6; Supplementary Table 8). Increased colony BQCV levels were positively correlated with weight while higher mite levels were associated with a decrease in weight (Figure 4; Supplementary Figure 2). While not significant (*P* = 0.06), both forager and colony DWV-B levels appeared to potentially interact with weight but with opposite directionality (Supplementary Table 8). As with individually foraged pollen P:L ratios, individually foraged nectar weight appeared to interact with bee stock in terms of correlations with colony viral infections (Supplementary Figures 7–9), requiring further investigation.

**Table 6:** Individual nectar forager generalized regression model effect test summaries for the nectar weight (mg) as well as the nectar sugar content (Brix, categorized into low, medium, and high values for an ordered logit).

		**Nectar weight**				**Sugar content**		
**Type**	**Variable**	**DF**	**Sum squares**	***F***	***P***	**DF**	**Wald χ^2^**	***P***
Forager	Virus no.	1	3.511	0.069	0.794	1	3.618	0.057
	BQCV	1	47.275	0.927	0.341	1	3.620	0.057
	CBPV	1	2.942	0.058	0.811	1	3.064	0.080
	DWV-A	1	0.005	0.000	0.992	1	0.013	0.908
	DWV-B	1	190.322	3.732	0.060	1	0.043	0.836
Colony	Mites	1	257.840	5.056	0.030	1	2.644	0.104
	Virus no.	1	15.436	0.303	0.585	1	1.554	0.213
	BQCV	1	482.454	9.460	0.004	1	2.470	0.116
	CBPV	1	159.053	3.119	0.085	1	0.819	0.365
	DWV-A	1	1.405	0.028	0.869	1	0.302	0.583
	DWV-B	1	189.981	3.725	0.060	1	6.221	0.013

##### Sugar Content (Brix)

Sugar content (Brix) was without categorization ranged from 21.6 to 85% with a mean of 56.27 ± 3.45. No variables aside from nectar weight were significantly associated (using Spearman's ρ) with sugar content as a continuous variable (Table 4). The only variable of potential note was colony DWV-B, with a marginal association (*P* = 0.066). Since sugar content measurements were artificially bounded at 85%, regressions used low (0–33.0%), medium (33.1–66.0%), and high (66.1–85%) categories as the dependent variable. The categories had an observed probability of 0.223 (low), 0.370 (medium), and 0.407 (high). The sugar content regression model using ordered logit, indicated that only colony DWV-B levels were significant (Table 6; Supplementary Table 8), with higher colony DWV-B levels being positively associated with nectar sugar content (Supplementary Figures 3, 4).

### Relative Contribution Individual vs. Colony Infection

#### Pollen Foraging

When we compared the regression models with and without forager and colony virus panel data (Table 7), models with only colony variables had a higher *R*^2^ but also higher AICc and BIC values compared to the forager only models. For each aspect of pollen foraging, the full models with both forager and colony variables exhibited the best combination performance (in terms of *R*^2^, AICc, and BIC), indicating that a combination of forager and colony virus levels was impacting protein content of individually foraged pollen.

**Table 7:** Pollen model selection criteria comparing the full model with individual virus and colony virus metrics using several selection criteria.

**Forage**	**Model**	**Variables**	**-Log likelihood**	**AICc**	**BIC**	***R* ^2^**	***Adj. R* ^2^**
Protein (% DW)	Full	Month + i_virus + i_BQCV + i_CBPV + i_DWV-A + i_DWV-B + c_mites + c_virus + c_BQCV + c_CBPV + c_DWV-A + c_DWV-B	1,050.083	2,131.724	2,186.876	0.268	0.238
	Forager	Month + i_virus + i_BQCV + i_CBPV + i_DWV-A + i_DWV-B	1,050.933	2,120.439	2,153.892	0.264	0.248
	Colony	Month + c_mites + c_virus + c_BQCV + c_CBPV + c_DWV-A + c_DWV-B	1,939.220	3,898.796	3,942.898	0.295	0.286
Lipid (% DW)	Full	Month + i_virus + i_BQCV + i_CBPV + i_DWV-A + i_DWV-B + c_mites + c_virus + c_BQCV + c_CBPV + c_DWV-A + c_DWV-B	394.389	819.912	858.832	0.167	0.094
	Forager	Month + i_virus + i_BQCV + i_CBPV + i_DWV-A + i_DWV-B	400.390	817.809	840.812	0.098	0.059
	Colony	Month + c_mites + c_virus + c_BQCV + c_CBPV + c_DWV-A + c_DWV-B	783.687	1,586.002	1,618.586	0.124	0.103
P:L ratio	Full	Month + i_virus + i_BQCV + i_CBPV + i_DWV-A + i_DWV-B + c_mites + c_virus + c_BQCV + c_CBPV + c_DWV-A + c_DWV-B	431.248	893.630	932.551	0.155	0.081
	Forager	Month + i_virus + i_BQCV + i_CBPV + i_DWV-A + i_DWV-B	438.394	893.817	916.820	0.070	0.031
	Colony	Month + c_mites + c_virus + c_BQCV + c_CBPV + c_DWV-A + c_DWV-B	827.646	1,673.935	1,706.321	0.105	0.083

**Figure 5:**
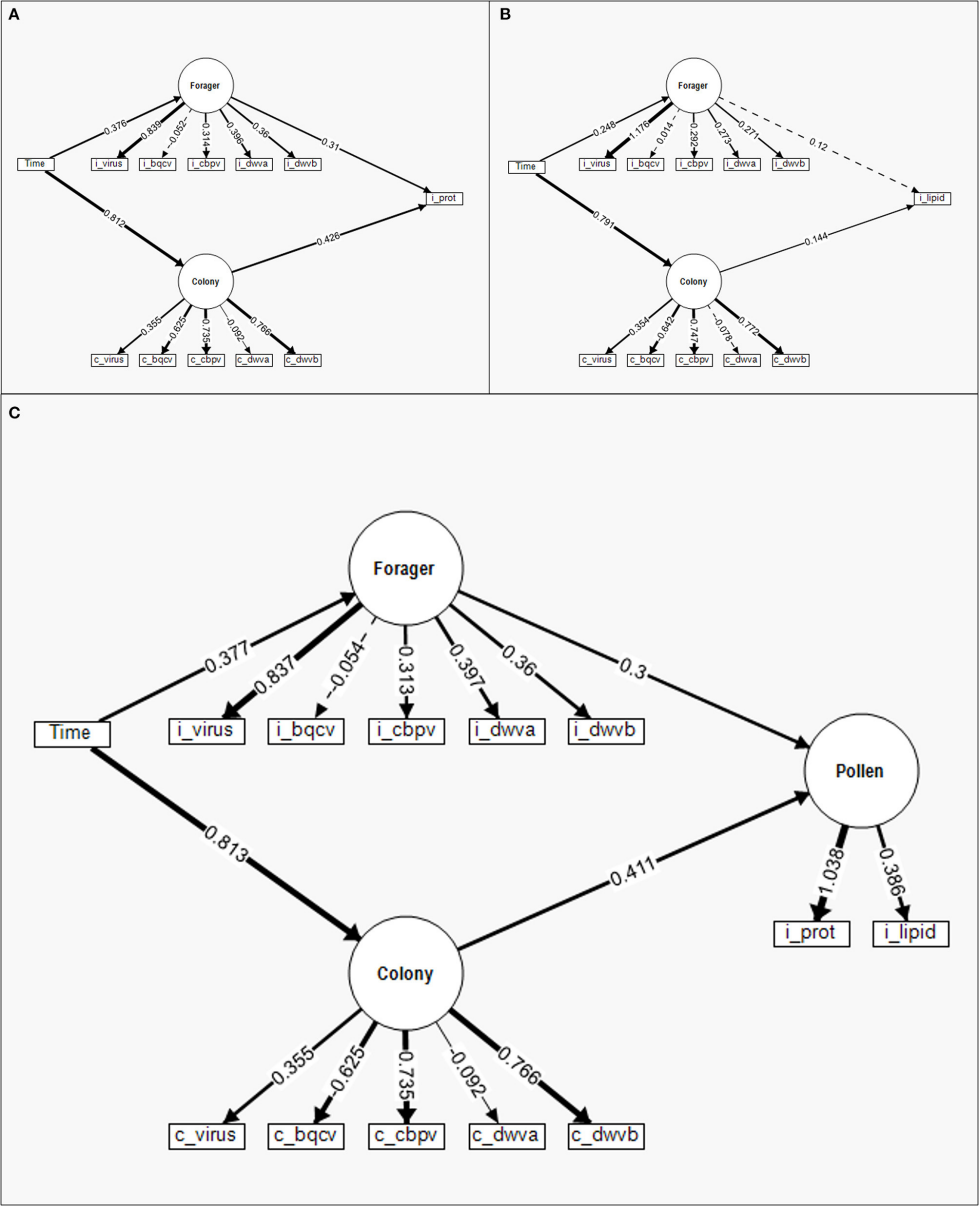
Structural equation models (SEM) to compare the standardized relative contribution of colony-level (c) disease (Colony) and individual (i) forager disease (Forager) to individual pollen foraging for **(A)** protein content, **(B)** lipid content, and **(C)** a latent variable combining protein and lipid content. Measured manifest variables are represented by boxes while latent factors are indicated by circles. Regression parameter estimates (given numbers) are standardized for comparison of variable contribution and presented in relation to the directed link (arrows) between variables. Solid lines indicate significant effects while dashed lines are insignificant. The line density indicates the relationship strength while the ± indicate the directionality of the relationship.

The structural equation model (SEM) analyzing the relative contributions of individual pollen forager viral infection and colony viral infection to individual pollen foraging on protein was relatively strong (χ^2^ = 670.793, CFI = 0.677, RMSEA = 0.137). The SEM (Figure 5A) indicated that the Colony (Wald *Z* = −9.336, *P* < 0.001) and Forager latent factors (Wald *Z* = 4.851, *P* = 0.327) had significant impacts on the individually foraged pollen protein content. Like the regression comparison, while both Forager and Colony factors contributed to individual pollen foraging decisions, the Colony factors contributed to a greater degree than Forager factors. Time had a significant effect on Colony (Wald *Z* = −38.213, *P* < 0.001) and Forager latent factors (Wald *Z* = 5.379, *P* < 0.001), with a greater relative impact on Colony. Further, the Colony and Forager latent factors significantly covaried (Wald *Z* = −3.282, *P* < 0.001), indicating the virus levels of one impacted the other (though directionality was not tested in this model). The SEM for lipid content (Figure 5B) was similarly strong (χ^2^ = 649.554, CFI = 0.656, RMSEA = 0.135). Unlike the protein SEM, the lipid SEM indicated that the Colony latent factors (Wald *Z* = 2.468, *P* = 0.014) but not the Forager latent factors (Wald *Z* = 1.552, *P* = 0.121) contributed to individually foraged lipid content. Time impacted both Colony (Wald *Z* = 36.596, *P* < 0.001) and Forager latent factors (Wald *Z* = 3.897, *P* < 0.001), with time having a larger impact on Colony factors than Forager factors. Colony and Forager latent factors again significantly covaried (Wald *Z* = −2.257, *P* = 0.024).

The combined protein and lipid SEM (Figure 5C) had model strength in line with the two component SEMS (χ^2^ = 701.015, CFI = 0.679, RMSEA = 0.127). In this model, the protein and lipid content of individually foraged pollen was combined into one Pollen latent factor. The results of this model were similar to prior model comparisons (Table 3), where both Colony (Wald *Z* = 6.597, *P* < 0.001) and Forager latent factors (Wald *Z* = 4.171, *P* < 0.001) significantly contributed to the Pollen latent factor, with Colony contributing to a greater extent. As with the prior two SEMs, time significantly impacted Colony (Wald *Z* = 38.187, *P* < 0.001) and Forager (Wald *Z* = 5.387, *P* < 0.001) latent factors with a greater impact on Colony. Additionally, Colony and Forager latent factors significantly covaried (Wald *Z* = −3.296, *P* = 0.001).

#### Nectar Foraging

The nectar regression models with and without forager and colony virus panel data (Table 8) indicated that the models with only colony variables had both the higher *R*^2^ and the lower AICc and BIC values compared to the forager only models. However, the full models with both forager and colony variables still exhibited the best combination performance (in terms of *R*^2^, AICc, and BIC).

**Table 8:** Nectar model selection criteria comparing the full model with individual virus and colony virus metrics using several selection criteria.

**Forage**	**Model**	**Variables**	**-Log likelihood**	**AICc**	**BIC**	***R* ^2^**	***Adj. R* ^2^**
Nectar weight (mg)	Full	i_virus + i_BQCV + i_CBPV + i_DWV-A + i_DWV-B + c_mites + c_virus + c_BQCV + c_CBPV + c_DWV-A + c_DWV-B	175.995	387.092	403.847	0.444	0.298
	Forager	i_virus + i_BQCV + i_CBPV + i_DWV-A + i_DWV-B	187.358	391.152	402.640	0.153	0.065
	Colony	c_mites + c_virus + c_BQCV + c_CBPV + c_DWV-A + c_DWV-B	178.978	377.157	389.869	0.379	0.300
Sugar content (Brix)	Full	i_virus + i_BQCV + i_CBPV + i_DWV-A + i_DWV-B + c_mites + c_virus + c_BQCV + c_CBPV + c_DWV-A + c_DWV-B	49.497	134.094	150.851	0.296	N/A
	Forager	i_virus + i_BQCV + i_CBPV + i_DWV-A + i_DWV-B	56.084	128.603	140.091	0.064	N/A
	Colony	c_mites + c_virus + c_BQCV + c_CBPV + c_DWV-A + c_DWV-B	52.859	124.917	137.629	0.185	N/A

*Adjusted R^2^ were not available for the sugar content model as it was an ordered logit*.

**Figure 6:**
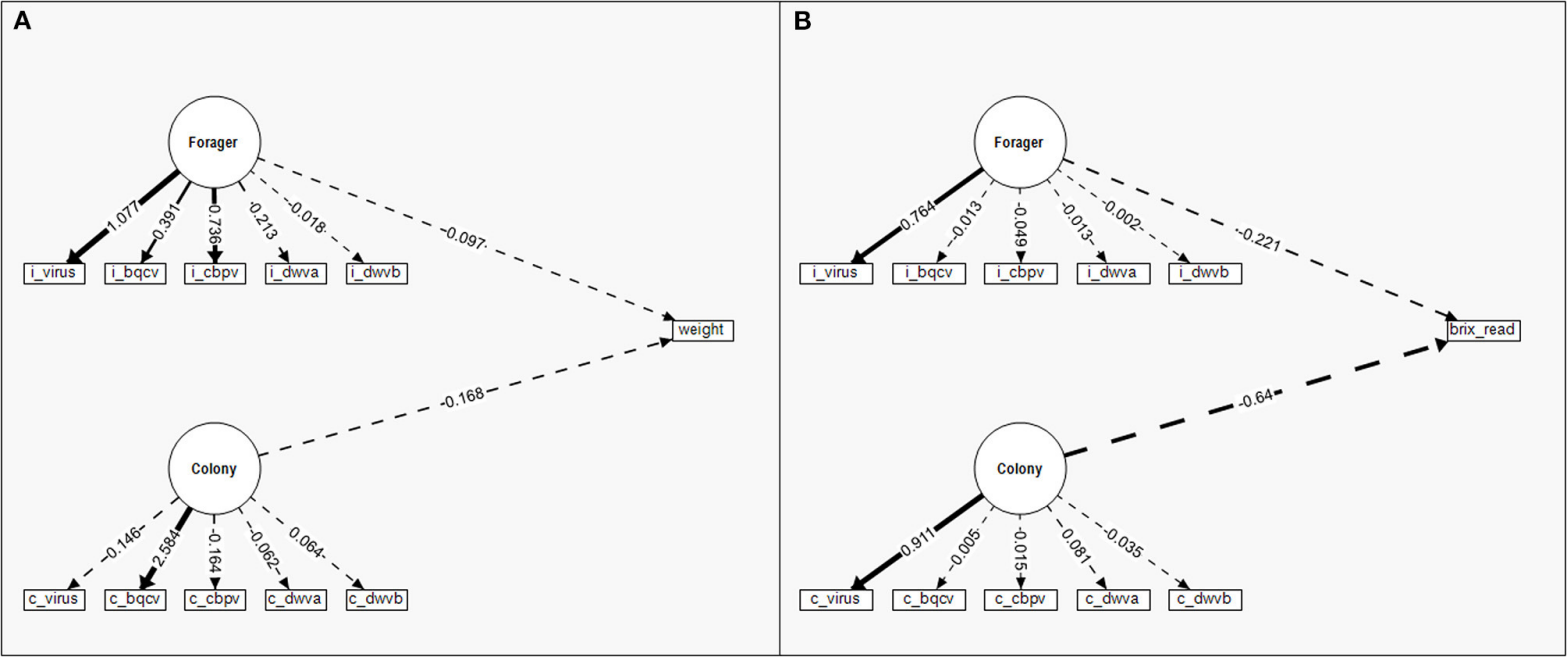
Structural equation models (SEM) to compare the standardized relative contribution of colony-level (c) disease (Colony) and individual (i) forager disease (Forager) to individual nectar foraging for **(A)** nectar weight and **(B)** nectar sugar content (Brix readings rather than categories as used in the regression). Measured manifest variables are represented by boxes while latent factors are indicated by circles. Regression parameter estimates (given numbers) are standardized for comparison of variable contribution and presented in relation to the directed link (arrows) between variables. Solid lines indicate significant effects while dashed lines are insignificant. The line density indicates the relationship strength while the ± indicate the directionality of the relationship.

The SEM for nectar weight (mg) (Figure 6A) was a weaker model than any of the pollen SEMS (χ^2^ = 146.942, CFI = 0.479, RMSEA = 0.215). Unlike the pollen SEMs, neither Colony (Wald *Z* = 1.032, *P* = 0.302) nor Forager (Wald *Z* = 0.796, *P* = 0.426) latent factors significantly influenced nectar weight. Colony and Forager latent factors also did not covary in the nectar weight SEM (Wald *Z* = 0.827, *P* = 0.408). The SEM for nectar sugar content (Brix) (Figure 6B) had similarly poor model strength (χ^2^ = 573.549, CFI = 0, RMSEA = 0.484). As with the nectar weight SEM, neither Colony (Wald *Z* = −1.078, *P* = 0.281) nor Forager (Wald *Z* = −0.586, *P* = 0.558) latent factors significantly influenced nectar sugar content. However, with the sugar content SEM, Colony and Forager latent factors did covary (Wald *Z* = 2.940, *P* = 0.003). When nectar weight and sugar content were combined into a single Nectar latent factor, the resulting model failed to converge under 1,000 model iterations and was not used.

## Discussion

It has been previously shown that honey bee selectively foraging on plant resources (such as pollen, nectar or resin) is related to the reduction of infection rates (9, 59, 94, 95). Pollen and nectar, in particular, assist in regulating bee immune response by impacting vitellogenin, detoxification, and metabolism-related gene expression, enabling the bee to maintain functionality (8, 11–13). Furthermore, the nutritional profile of the pollen can itself aid in survival after infection (57) since protein helps repair and build tissues while lipids provide energy for bees (12, 38). Recent evidence has also shown that certain natural plant products may reduce impacts of viral infection (96). Our study evaluated the protein and lipid content of individually foraged pollen as well as the weight and sugar content of collected nectar in relation to honey bee viral infection. Further, we investigated whether these interactions at the individual forager level were based primarily on colony or individual forager viral infections.

### Interactions of Disease and Foraging

The range of bee viruses and foraged pollen macronutrient profiles from our study generally matched that of previous studies. Most colonies tested positive for a suite of viruses—ABPV, BQCV, CBPV, DWV-A, DWV-B, IAPV, and LSV—similar to previous studies (97–99). The overall range of colony-foraged pollen protein (Range = 10.5–18.4%) and lipid contents (Range = 0.7–3.14%) were within the range of prior work on US honey bees (100). The variation in individually foraged pollen protein (Range 0.03–33.95%) and lipid contents (Range = 0.095–15.384%) was greater than in colony-collected pollen. This is expected as each forager sample may represent pollen from a single plant species targeted by that individual on a single day (101, 102), while the colony sample was an amalgamation of individual samples over 4 days. Given that all colonies were based within the same apiary, colonies had an equal opportunity to forage from the same floral resources (103) and differences were due in colony-level variation such as viral infection (6, 46–48).

The first goal of this work was to determine if *Varroa* mites and viral loads were correlated with differences in pollen macronutrient (protein and lipid) and nectar foraging. Prior work has shown that high-protein pollen can increase honey bee survival when faced with infection while high-lipid diets may increase susceptibility to pathogens (41, 57, 72). Therefore, we expected that higher mite and viral loads would generally increase foraging on higher-protein pollen. With the Spearman's correlation we found that higher mite numbers were positively associated with pollen protein content, but no association was found using generalized regression. However, the mite loads in our colonies remained relatively low throughout the study and may not have been variable enough to obtain the most reliable data in this regard. The viruses differed in their correlation with individually foraged pollen protein and lipid contents based on the type of virus and if the individual or colony was infected. The correlations and regressions indicated that colony DWV-B levels were negatively associated with the pollen P:L ratio and positively with lipid content, whereas the total number of viruses at the colony level was negatively associated with lipid content and positively with P:L ratios.

In addition to altering pollen foraging preferences, microbial infections have the potential to change bees' ability to perceive sugar content and nectar foraging behaviors (37, 67, 104, 105). While we expected that higher viral loads would also be associated with an increase in nectar sugar content, we found that this was only the case with colony DWV-B infections. Nectar load weights also significantly increased with higher colony BQCV levels and marginally increased with higher colony DWV-B levels, another indication that different stressors may induce differential foraging (53, 56). However, individual forager DWV-B infections tended (though marginally) to decrease nectar load weight, indicating some tradeoff of individual ability and colony health (47, 67).

These foraging interactions based on virus type suggest differential immune responses and subsequent nutritional requirements in response to different viruses (106–109). For instance, honey bee transcriptional and immune responses differ greatly from bacteria and *Nosema* to viral infections and between viral infections such as IAPV and DWV (110, 111). Further, energetic costs to honey bees differs with parasite or pathogen identity (104, 108). Work by Annoscia et al. (38) indicated that bees infested with *Varroa* mites, often associated with DWV levels, and fed pollen diets had up-regulated genes related to lipid metabolism; whereas, Rutter et al. (112) indicated that IAPV interacted with diet *via* carbohydrate metabolism-related genes. The impact of diet on immune function in the face of viral stressors may also vary with time of year and gut microbiota community structure (113–115).

Not only does diet alter honey bee ability to overcome viral challenges, but it also alters honey bee physiological responses to another stressor—pesticides. Individual bees fed lower P:L diets exhibited longer lifespans than those fed high P:L diets when both sets were exposed to pesticides, potentially due to these diets also altering expression of genes like Vg (vitellogenin) and Defensin-1 (49). Viruses and *Varroa* infestation can interact with pesticide exposure to make bees more susceptible to the other stressor; though the strength of these interactions depends on the combination of pesticide and virus (116). *Varroa* mite infestation and DWV infection increase bee susceptibility to insecticides and vice versa (117, 118). Similarly, thiamethoxam exposure increased CBPV loads (119), and thiacloprid increased BQCV viral loads in honey bees (120). The interactions of pesticides and viral infection may be due to how both types of stressors impact bee immune response and gene expression (116, 121). This is demonstrated particularly well by how winter vs. summer bees respond to either stressor—winter bees are generally more susceptible to both stressors and have lower immune gene expression than summer bees (113, 115). Similarly, colonies in agricultural landscapes (where they are likely to encounter pesticides) with access to more floral resources in the summer, have higher Vg expression and greater winter survival rates than colonies with access to poorer food resources in the summer (122). This highlights the need to study the complex interactions of multiple stressors and the potential benefits of increased access to high quality nutrition or more targeted nutritional supplement strategies based on colony states or environmental pressures.

### Importance of Collective Foraging

Bees are known to adjust gross foraging preferences based on the demands of the colony and social immunity requirements (66, 123, 124). Therefore, the second aspect of this study was to determine if individual honey bee foragers selected food items based primarily on their individual or colony's viral infections. Our data indicate that despite the tensions between individual benefits and social immunity (47, 63), the impact of colony viral infections appeared to outweigh individual infection in both the SEMs and regression comparisons. This may be due, in part, to the differences in infection levels between foragers and colonies—forager viral infections except for BQCV had lower levels and were less variable than those found in the colony-representative nurse bee samples, meaning that nurse bee infections may be more acute or there is survival bias by the time bees become foragers. So, while individual forager infection might reduce foraging efficiency (125), collective foraging decisions are directed by social immunity needs.

The importance of social immunity in collective foraging decisions is also compatible with prior observations of collective foraging of forager groups being dispatched to several food patches rather than individuals comparing food quality across patches (15, 24, 126, 127). These foragers make individual decisions about a food patch that depend on nutritional content and availability of food items that then accumulate across foragers for a collective outcome (128). However, if collective foraging is impacted by colony viral infection, individual foragers must be able to detect shifts in colony viral loads. This may occur *via* infection or parasitism-induced alterations to the bee cuticular hydrocarbon profile, signaling to nestmates (like foragers) changes in health status that may result in nestmate (forager) behavioral changes (129–132). For instance, in colonies with high *Varroa* mite loads, foragers changed dancing locations to be closer to the frame periphery relative to colonies with lower mite levels (133). Like the overall colony need for food resources, changes in chemical cues based on infection and/or parasitism may indicate to foragers that macronutrient preferences need to be adjusted.

Since several of the viruses found to influence bee foraging are vectored by *Varroa* mites (134, 135), we expected to potentially observe foraging differences based on the interaction of mite resistant bee stocks and viruses (52, 136, 137). Preliminary data (Figures 3, 4; Supplementary Figures 1, 2, 5–9) indicate that two mite-resistant stocks may be more responsive to viral infection in terms of P:L ratios and nectar load weights than the susceptible Italian stock. However, the mite resistant stocks did not appear to fully align with each other in how and for which viruses they changed their foraging. Given that the mechanism of mite resistance differs between Pol-Line and Russian stocks (136, 137), we might expect that the two genotypes also differ in their immune response and related nutritional requirements when infected with mite-vectored viruses (138). While preliminary due to low colony sample sizes per stock, these observations indicate that overall mite resistance may influence the host's likelihood to forage in response to pathogens (86, 103, 139, 140) but that the genetic separation of mite-resistant genotypes may further differentiate responses to viruses (52, 141). The incorporation of host genetics into future investigations of host-pathogen interactions is critical for understanding more global effects of pathogen challenges, particularly given the potential of *Varroa* mites becoming resistant to current miticides and in relation to mite-vectored viruses (142–146).

## Conclusion

Our observational field study evaluated foraging preferences associated with differences in naturally occurring viral infection levels between colonies and individual foragers with preliminary data across bee stocks with varying resistance to *Varroa* mites. Overall, we found that higher colony virus numbers generally decreased lipid foraging, with levels of viruses like DWV-B exhibiting differential interactions that may further interact with mite-resistant bee stocks. *Varroa* mite levels also decreased nectar load weights while select virus levels (BQCV and DWV-B) increased load weight and sugar content, respectively. When we evaluated the relative importance of colony and individual viral loads on individual foraging, we found that foragers exhibit preferences based primarily on colony virus loads. Taken together, these data indicate differential foraging responses for different viral infections and collective foraging may be an important aspect of social immunity in honey bees. Overall, this work highlights the continued need to explore the combined and complex interactions among disease, nutrition, and genetics in social networks.

## Data Availability Statement

The raw data supporting the conclusions of this article will be made available by the authors, without undue reservation.

## Author Contributions

HP, MS-F, and LG: conceptualization, methodology, and funding acquisition. RD, HP, and MS-F: formal analysis. RD, PT, HP, and LG: investigation. MS-F and LG: resources. RD, PT, and MS-F: validation. RD and HP: data curation. HP: writing—original draft preparation and visualization. PT, RD, MS-F, and LG: writing—review and editing. MS-F: supervision. RD: project administration. All authors have read and agree to the published version of the manuscript.

## Funding

This research was funded by Project Apis m. and the National Honey Board Grant Number 58-6050-9-001, the Louisiana Beekeepers Association, and the USDA ARS.

## Author Disclaimer

Mention of trade names or commercial products in this publication is solely for the purpose of providing specific information and does not imply recommendation or endorsement by the U.S. Department of Agriculture. USDA is an equal opportunity provider and employer.

## Conflict of Interest

The authors declare that the research was conducted in the absence of any commercial or financial relationships that could be construed as a potential conflict of interest.

## Publisher's Note

All claims expressed in this article are solely those of the authors and do not necessarily represent those of their affiliated organizations, or those of the publisher, the editors and the reviewers. Any product that may be evaluated in this article, or claim that may be made by its manufacturer, is not guaranteed or endorsed by the publisher.
