# Supplementary material for: Colony-Level Viral Load Influences Collective Foraging in Honey Bees
Source: Front Insect Sci. 2022 May 17;2:894482. doi: 10.3389/finsc.2022.894482 (PMC10926460; doi:10.3389/finsc.2022.894482)

**Table S1.** *Colony virus* *prevalence*: Whether (+) or not (-) each colony tested positive (Ct < 40 cycles) for each viral infection at each time from a pooled sampled of 50 bees collected from a brood frame.

| **Time** | **Stock** | **Colony** | **ABPV** | **BQCV** | **CBPV** | **DWV-A** | **DWV-B** | **IAPV** | **KBV** | **LSV** |
| --- | --- | --- | --- | --- | --- | --- | --- | --- | --- | --- |
| **1: July** | *Italian* | *209* | + | + | + | + | + | - | - | + |
|  |  | *227* | + | + | - | + | + | - | + | + |
|  |  | *233* | + | + | - | + | + | - | - | + |
|  | *Pol-Line* | *207* | + | + | - | + | + | - | - | + |
|  |  | *216* | + | + | - | + | + | - | - | + |
|  |  | *224* | + | + | - | + | + | - | - | + |
|  | *Russian* | *211* | + | + | - | + | + | - | - | + |
|  |  | *218* | + | + | - | + | + | - | - | + |
|  |  | *TQ19-21* | + | + | - | + | + | - | - | + |
| **2: Aug.** | *Italian* | *209* | + | + | + | + | + | + | - | + |
|  |  | *227* | + | + | - | + | + | + | - | + |
|  |  | *233* | + | + | + | + | + | + | - | + |
|  | *Pol-Line* | *207* | + | + | - | + | + | + | - | + |
|  |  | *216* | + | + | - | + | + | + | - | + |
|  |  | *224* | + | + | - | + | + | + | - | + |
|  | *Russian* | *211* | + | + | - | + | + | + | - | + |
|  |  | *218* | + | + | - | + | + | + | - | + |
|  |  | *TQ19-21* | + | + | + | + | + | + | - | - |
| **3: Sept.** | *Italian* | *209* | + | + | + | + | + | + | - | + |
|  |  | *227* | + | + | + | + | + | + | - | - |
|  |  | *233* | + | + | + | + | + | + | - | + |
|  | *Pol-Line* | *207* | + | + | + | + | + | + | - | + |
|  |  | *216* | + | + | - | + | + | + | - | + |
|  |  | *224* | + | + | + | + | + | + | - | + |
|  | *Russian* | *211* | + | + | + | + | + | + | - | + |
|  |  | *218* | + | + | + | + | + | + | - | + |
|  |  | *TQ19-21* | + | + | - | + | + | + | - | + |

**Table S2.** *Colony virus* *loads*: The log-transformed virus load (using a pooled sampled of 50 bees collected from a brood frame/colony/time) for each colony at each time.

| **Time** | **Stock** | **Colony** | **BQCV** | **CBPV** | **DWV-A** | **DWV-B** |
| --- | --- | --- | --- | --- | --- | --- |
| **1: July** | *Italian* | *209* | 4.599 | 4.579 | 4.950 | 8.310 |
|  |  | *227* | 5.507 | 0.000 | 7.638 | 9.380 |
|  |  | *233* | 5.142 | 0.000 | 14.061 | 9.260 |
|  | *Pol-Line* | *207* | 4.011 | 0.000 | 10.647 | 5.711 |
|  |  | *216* | 4.065 | 0.000 | 10.523 | 5.336 |
|  |  | *224* | 7.429 | 0.000 | 5.033 | 7.005 |
|  | *Russian* | *211* | 4.614 | 0.000 | 4.456 | 6.042 |
|  |  | *218* | 5.069 | 0.000 | 10.557 | 5.493 |
|  |  | *TQ19-21* | 6.674 | 0.000 | 8.661 | 6.181 |
| **2: Aug.** | *Italian* | *209* | 4.171 | 4.378 | 8.757 | 9.436 |
|  |  | *227* | 4.454 | 0.000 | 8.145 | 9.588 |
|  |  | *233* | 5.289 | 4.489 | 7.952 | 9.964 |
|  | *Pol-Line* | *207* | 4.298 | 0.000 | 3.922 | 9.139 |
|  |  | *216* | 5.802 | 0.000 | 7.279 | 5.500 |
|  |  | *224* | 4.333 | 0.000 | 8.920 | 9.265 |
|  | *Russian* | *211* | 3.837 | 0.000 | 3.717 | 7.658 |
|  |  | *218* | 6.259 | 0.000 | 4.496 | 7.729 |
|  |  | *TQ19-21* | 3.929 | 4.257 | 8.300 | 13.801 |
| **3: Sept.** | *Italian* | *209* | 4.364 | 5.379 | 9.659 | 8.267 |
|  |  | *227* | 3.887 | 4.419 | 5.781 | 11.020 |
|  |  | *233* | 4.545 | 4.280 | 8.969 | 9.268 |
|  | *Pol-Line* | *207* | 4.194 | 4.387 | 10.235 | 12.883 |
|  |  | *216* | 3.925 | 0.000 | 4.921 | 11.458 |
|  |  | *224* | 3.877 | 4.364 | 7.030 | 8.946 |
|  | *Russian* | *211* | 4.025 | 4.261 | 7.603 | 9.624 |
|  |  | *218* | 5.159 | 6.358 | 5.205 | 12.883 |
|  |  | *TQ19-21* | 3.795 | 0.000 | 6.554 | 10.600 |

**Table S3.** *Individual pollen forager virus* *prevalence*: The number of bees out of a total possible of 12 bees testing positive (Ct < 40 cycles) for each viral infection at each time (N = 12 bees/colony/sampling time).

| **Time** | **Stock** | **Colony** | **ABPV** | **BQCV** | **CBPV** | **DWV-A** | **DWV-B** | **IAPV** | **KBV** | **LSV** |
| --- | --- | --- | --- | --- | --- | --- | --- | --- | --- | --- |
| **1: July** | *Italian* | *209* | 0 | 12 | 0 | 12 | 12 | 11 | 0 | 6 |
|  |  | *227* | 2 | 12 | 0 | 11 | 7 | 9 | 0 | 1 |
|  |  | *233* | 3 | 12 | 4 | 12 | 12 | 10 | 0 | 3 |
|  | *Pol-Line* | *207* | 6 | 12 | 5 | 9 | 12 | 7 | 0 | 3 |
|  |  | *216* | 3 | 12 | 2 | 4 | 12 | 8 | 0 | 7 |
|  |  | *224* | 7 | 12 | 0 | 12 | 12 | 12 | 4 | 4 |
|  | *Russian* | *211* | 5 | 12 | 2 | 9 | 4 | 11 | 2 | 1 |
|  |  | *218* | 1 | 12 | 0 | 12 | 12 | 12 | 0 | 6 |
|  |  | *TQ19-21* | 1 | 12 | 1 | 11 | 12 | 10 | 1 | 8 |
| **2: Aug.** | *Italian* | *209* | 7 | 12 | 0 | 9 | 12 | 12 | 0 | 4 |
|  |  | *227* | 2 | 12 | 0 | 11 | 12 | 12 | 0 | 3 |
|  |  | *233* | 8 | 12 | 1 | 8 | 12 | 12 | 0 | 3 |
|  | *Pol-Line* | *207* | 9 | 12 | 2 | 12 | 12 | 12 | 1 | 1 |
|  |  | *216* | 11 | 12 | 6 | 7 | 12 | 11 | 0 | 3 |
|  |  | *224* | 4 | 12 | 8 | 9 | 12 | 12 | 0 | 7 |
|  | *Russian* | *211* | 3 | 12 | 11 | 6 | 12 | 11 | 1 | 2 |
|  |  | *218* | 4 | 12 | 1 | 12 | 12 | 11 | 0 | 7 |
|  |  | *TQ19-21* | 1 | 12 | 1 | 12 | 12 | 12 | 0 | 7 |
| **3: Sept.** | *Italian* | *209* | 10 | 12 | 0 | 12 | 12 | 12 | 3 | 7 |
|  |  | *227* | 11 | 12 | 1 | 12 | 9 | 12 | 3 | 7 |
|  |  | *233* | 9 | 12 | 1 | 12 | 12 | 12 | 1 | 8 |
|  | *Pol-Line* | *207* | 12 | 12 | 4 | 11 | 4 | 10 | 0 | 2 |
|  |  | *216* | 11 | 12 | 6 | 11 | 2 | 12 | 0 | 2 |
|  |  | *224* | 6 | 12 | 0 | 12 | 12 | 12 | 0 | 4 |
|  | *Russian* | *211* | 7 | 12 | 0 | 12 | 12 | 12 | 0 | 3 |
|  |  | *218* | 9 | 12 | 3 | 11 | 12 | 11 | 0 | 4 |
|  |  | *TQ19-21* | 5 | 12 | 1 | 12 | 12 | 12 | 2 | 7 |

**Table S4.** *Individual pollen forager virus* *loads*: The average log-transformed virus load ± standard error of the mean (N = 12 bees/colony/time) for each viral infection at each time.

| **Time** | **Stock** | **Colony** | **BQCV** | **CBPV** | **DWV-A** | **DWV-B** |
| --- | --- | --- | --- | --- | --- | --- |
| **1: July** | *Italian* | *209* | 5.686 ± 0.21 | 0.000 | 4.617 ± 0.13 | 7.452 ± 0.55 |
|  |  | *227* | 6.139 ± 0.32 | 0.000 | 4.379 ± 0.19 | 6.543 ± 0.90 |
|  |  | *233* | 5.874 ± 0.27 | 1.450 ± 0.62 | 4.564 ± 0.10 | 7.253 ± 0.61 |
|  | *Pol-Line* | *207* | 6.109 ± 0.27 | 1.278 ± 0.55 | 3.546 ± 0.16 | 5.537 ± 0.09 |
|  |  | *216* | 5.780 ± 0.23 | 0.678 ± 0.46 | 3.183 ± 0.20 | 5.158 ± 0.15 |
|  |  | *224* | 6.556 ± 0.40 | 0.330 ± 0.33 | 5.509 ± 0.45 | 7.525 ± 0.62 |
|  | *Russian* | *211* | 6.067 ± 0.19 | 0.611± 0.41 | 4.017 ± 0.20 | 3.953 ± 0.31 |
|  |  | *218* | 6.181 ± 0.14 | 0.000 | 4.136 ± 0.40 | 5.738 ± 0.23 |
|  |  | *TQ19-21* | 5.719 ± 0.22 | 0.346 ± 0.35 | 4.693 ± 0.49 | 5.656 ± 0.10 |
| **2: Aug.** | *Italian* | *209* | 5.556 ± 0.12 | 0.000 | 4.039 ± 0.59 | 8.034 ± 0.68 |
|  |  | *227* | 5.490 ± 0.12 | 0.000 | 3.737 ± 0.11 | 8.118 ± 0.62 |
|  |  | *233* | 5.165 ± 0.12 | 0.326 ± 0.33 | 3.696 ± 0.23 | 8.678 ± 0.72 |
|  | *Pol-Line* | *207* | 5.582 ± 0.12 | 0.663 ± 0.45 | 3.857 ± 0.05 | 6.731 ± 0.37 |
|  |  | *216* | 5.252 ± 0.14 | 1.856 ± 0.66 | 3.275 ± 0.14 | 5.797 ± 0.15 |
|  |  | *224* | 6.755 ± 0.31 | 2.810 ± 0.61 | 4.068 ± 0.62 | 6.698 ± 0.73 |
|  | *Russian* | *211* | 5.688 ± 0.20 | 3.734 ± 0.36 | 3.123 ± 0.15 | 5.409 ± 0.16 |
|  |  | *218* | 5.563 ± 0.18 | 0.324 ± 0.32 | 3.790 ± 0.05 | 5.955 ± 0.06 |
|  |  | *TQ19-21* | 5.509 ± 0.18 | 0.661 ± 0.45 | 3.997 ± 0.04 | 6.124 ± 0.03 |
| **3: Sept.** | *Italian* | *209* | 5.084 ± 0.13 | 0.000 | 5.161 ± 0.24 | 8.872 ± 0.66 |
|  |  | *227* | 5.293 ± 0.17 | 0.324 ± 0.32 | 5.188 ± 0.23 | 7.004 ± 0.61 |
|  |  | *233* | 4.812 ± 0.08 | 0.332 ± 0.33 | 4.733 ± 0.12 | 10.179 ± 0.74 |
|  | *Pol-Line* | *207* | 5.878 ± 0.29 | 1.286 ± 0.55 | 4.903 ± 0.11 | 5.854 ± 1.04 |
|  |  | *216* | 5.390 ± 0.11 | 1.966 ± 0.59 | 4.483 ± 0.26 | 3.853 ± 0.54 |
|  |  | *224* | 5.552 ± 0.18 | 0.000 | 4.526 ± 0.07 | 6.094 ± 0.26 |
|  | *Russian* | *211* | 5.595± 0.23 | 0.000 | 4.407 ± 0.12 | 6.870 ± 0.42 |
|  |  | *218* | 5.129 ± 0.22 | 0.975 ± 0.51 | 4.165 ± 0.23 | 6.082 ± 0.16 |
|  |  | *TQ19-21* | 5.078 ± 0.14 | 0.317 ± 0.32 | 4.081 ± 0.07 | 6.532 ± 0.08 |

**Table S5.** *Individual nectar forager virus* *prevalence*: The number of bees out of a total possible 6 bees/colony testing positive (Ct < 40 cycles) for each viral infection on 7/25/2019.

| **Stock** | **Colony** | **ABPV** | **BQCV** | **CBPV** | **DWV-A** | **DWV-B** | **LSV** |
| --- | --- | --- | --- | --- | --- | --- | --- |
| **Italian** | *209* | 6 | 6 | 3 | 6 | 6 | 5 |
|  | *227* | 4 | 6 | 4 | 6 | 6 | 5 |
|  | *233* | 5 | 6 | 1 | 6 | 6 | 6 |
| **Pol-Line** | *207* | 6 | 6 | 4 | 6 | 6 | 6 |
|  | *216* | 5 | 6 | 3 | 5 | 6 | 6 |
|  | *224* | 6 | 6 | 5 | 6 | 6 | 6 |
| **Russian** | *211* | 5 | 5 | 4 | 6 | 6 | 6 |
|  | *218* | 3 | 6 | 4 | 6 | 6 | 5 |
|  | *TQ19-21* | 5 | 6 | 4 | 6 | 6 | 6 |

**Table S6.** *Individual nectar forager virus* *loads*: The average log-transformed virus load ± standard error of the mean (N = 6 bees/colony/time) for each colony on 7/25/2019.

| **Stock** | **Colony** | **BQCV** | **CBPV** | **DWV-A** | **DWV-B** |
| --- | --- | --- | --- | --- | --- |
| **Italian** | *209* | 6.164 ± 0.35 | 2.547 ± 1.14 | 5.649 ± 0.43 | 7.520 ± 0.30 |
|  | *227* | 6.427 ± 0.55 | 3.264 ± 1.04 | 5.785 ± 0.25 | 9.247 ± 1.00 |
|  | *233* | 5.589 ± 0.41 | 0.760 ± 0.76 | 5.210 ± 0.17 | 7.950 ± 0.89 |
| **Pol-Line** | *207* | 5.844 ± 0.21 | 3.382 ± 1.08 | 5.604 ± 0.22 | 8.007 ± 0.90 |
|  | *216* | 7.215 ± 0.47 | 2.525 ± 1.13 | 4.252 ± 0.87 | 7.048 ± 0.94 |
|  | *224* | 7.417 ± 0.47 | 4.023 ± 0.81 | 5.989 ± 0.17 | 7.040 ± 0.12 |
| **Russian** | *211* | 5.381 ± 1.19 | 3.129 ± 0.99 | 5.393 ± 0.13 | 6.536 ± 0.16 |
|  | *218* | 6.448 ± 0.39 | 3.075 ± 0.97 | 5.341 ± 0.18 | 6.495 ± 0.22 |
|  | *TQ19-21* | 5.177 ± 0.25 | 3.163 ± 1.00 | 5.497 ± 0.17 | 6.324 ± 0.12 |

**Table S7.** Individual pollen forager generalized regression model parameter estimates (relative to model intercept) for the percentage of protein and lipid in the collected pollen (dry weight) as well as the protein to lipid ratio (P:L).

|  | | Protein | | | | Lipid | | | | P:L | | | |
| --- | --- | --- | --- | --- | --- | --- | --- | --- | --- | --- | --- | --- | --- |
| Type | Variable | Estimate | St. Error | Wald χ² | *P* | Estimate | St. Error | Wald Chi-Square | *P* | Estimate | St. Error | Wald χ² | *P* |
| Intercept | Intercept | -0.291 | 6.575 | 0.002 | 0.965 | 7.787 | 5.476 | 2.023 | 0.155 | -1.163 | 7.012 | 0.027 | 0.868 |
| Month | August | 3.571 | 1.319 | 7.332 | 0.007 |  |  |  |  |  |  |  |  |
|  | September | 7.495 | 1.388 | 29.148 | 0.000 | 2.603 | 1.273 | 4.178 | 0.041 | 3.672 | 1.631 | 5.069 | 0.024 |
| Forager | Virus No. | 0.507 | 0.421 | 1.446 | 0.229 | 0.435 | 0.368 | 1.399 | 0.237 | 0.156 | 0.471 | 0.110 | 0.740 |
|  | BQCV | -0.379 | 0.478 | 0.630 | 0.428 | 0.437 | 0.423 | 1.064 | 0.302 | -0.042 | 0.542 | 0.006 | 0.938 |
|  | CBPV | 0.451 | 0.256 | 3.092 | 0.079 | 0.418 | 0.238 | 3.086 | 0.079 | -0.253 | 0.305 | 0.687 | 0.407 |
|  | DWV-A | 0.497 | 0.419 | 1.405 | 0.236 | -0.357 | 0.437 | 0.669 | 0.413 | 0.261 | 0.559 | 0.217 | 0.641 |
|  | DWV-B | 0.191 | 0.212 | 0.804 | 0.370 | 0.083 | 0.191 | 0.186 | 0.666 | -0.094 | 0.245 | 0.148 | 0.700 |
| Colony | Mites | -0.085 | 0.085 | 0.987 | 0.320 | -0.079 | 0.060 | 1.711 | 0.191 | 0.075 | 0.077 | 0.954 | 0.329 |
|  | Virus No. | -0.040 | 0.671 | 0.004 | 0.953 | -1.357 | 0.719 | 3.559 | 0.059 | 0.128 | 0.921 | 0.019 | 0.890 |
|  | BQCV | 0.233 | 0.546 | 0.182 | 0.669 | -0.064 | 0.448 | 0.020 | 0.887 | 0.613 | 0.574 | 1.140 | 0.286 |
|  | CBPV | 0.183 | 0.244 | 0.565 | 0.452 | -0.023 | 0.206 | 0.012 | 0.912 | 0.379 | 0.263 | 2.067 | 0.151 |
|  | DWV-A | 0.024 | 0.180 | 0.018 | 0.893 | -0.026 | 0.165 | 0.024 | 0.876 | 0.379 | 0.211 | 3.219 | 0.073 |
|  | DWV-B | 0.030 | 0.247 | 0.014 | 0.904 | -0.046 | 0.245 | 0.034 | 0.853 | -0.691 | 0.314 | 4.829 | 0.028 |

**Table S8.** Individual nectar forager generalized regression model parameter estimates (relative to model intercept) for nectar weight (mg) and sugar content (Brix, categorized into low, medium, and high values for an ordered logit where low=intercept1 and medium=intercept2).

|  |  | Nectar Weight | | | | Sugar Content | | | |
| --- | --- | --- | --- | --- | --- | --- | --- | --- | --- |
| Type | Variable | Estimate | St. Error | Wald χ² | *P* | Estimate | St. Error | Wald χ² | *P* |
| Intercept | Intercept:1 | -2.158 | 21.481 | 0.010 | 0.920 | -0.297 | 6.073 | 0.002 | 0.961 |
|  | Intercept:2 |  |  |  |  | 2.119 | 0.440 | 23.187 | 0.000 |
| Forager | Virus No. | -0.729 | 2.779 | 0.069 | 0.793 | -1.493 | 0.785 | 3.618 | 0.057 |
|  | BQCV | 0.764 | 0.794 | 0.927 | 0.336 | 0.473 | 0.249 | 3.620 | 0.057 |
|  | CBPV | -0.185 | 0.770 | 0.058 | 0.810 | 0.369 | 0.211 | 3.064 | 0.080 |
|  | DWV-A | -0.012 | 1.239 | 0.000 | 0.992 | -0.035 | 0.301 | 0.013 | 0.908 |
|  | DWV-B | -1.328 | 0.687 | 3.732 | 0.053 | 0.040 | 0.193 | 0.043 | 0.836 |
| Colony | Mites | -6.006 | 2.671 | 5.056 | 0.025 | -1.223 | 0.752 | 2.644 | 0.104 |
|  | Virus No. | -1.960 | 3.563 | 0.303 | 0.582 | -1.247 | 1.000 | 1.554 | 0.213 |
|  | BQCV | 3.764 | 1.224 | 9.460 | 0.002 | 0.531 | 0.338 | 2.470 | 0.116 |
|  | CBPV | 2.102 | 1.190 | 3.119 | 0.077 | 0.309 | 0.341 | 0.819 | 0.365 |
|  | DWV-A | 0.118 | 0.709 | 0.028 | 0.868 | -0.109 | 0.197 | 0.302 | 0.583 |
|  | DWV-B | 2.995 | 1.552 | 3.725 | 0.054 | 1.124 | 0.451 | 6.221 | 0.013 |

**Figure S1.** Protein: lipid ratios of individually foraged pollen relative to log-transformed forager levels of (A) BQCV, (B) CBPV, (C) DWV-A, and (D) DWV-B.


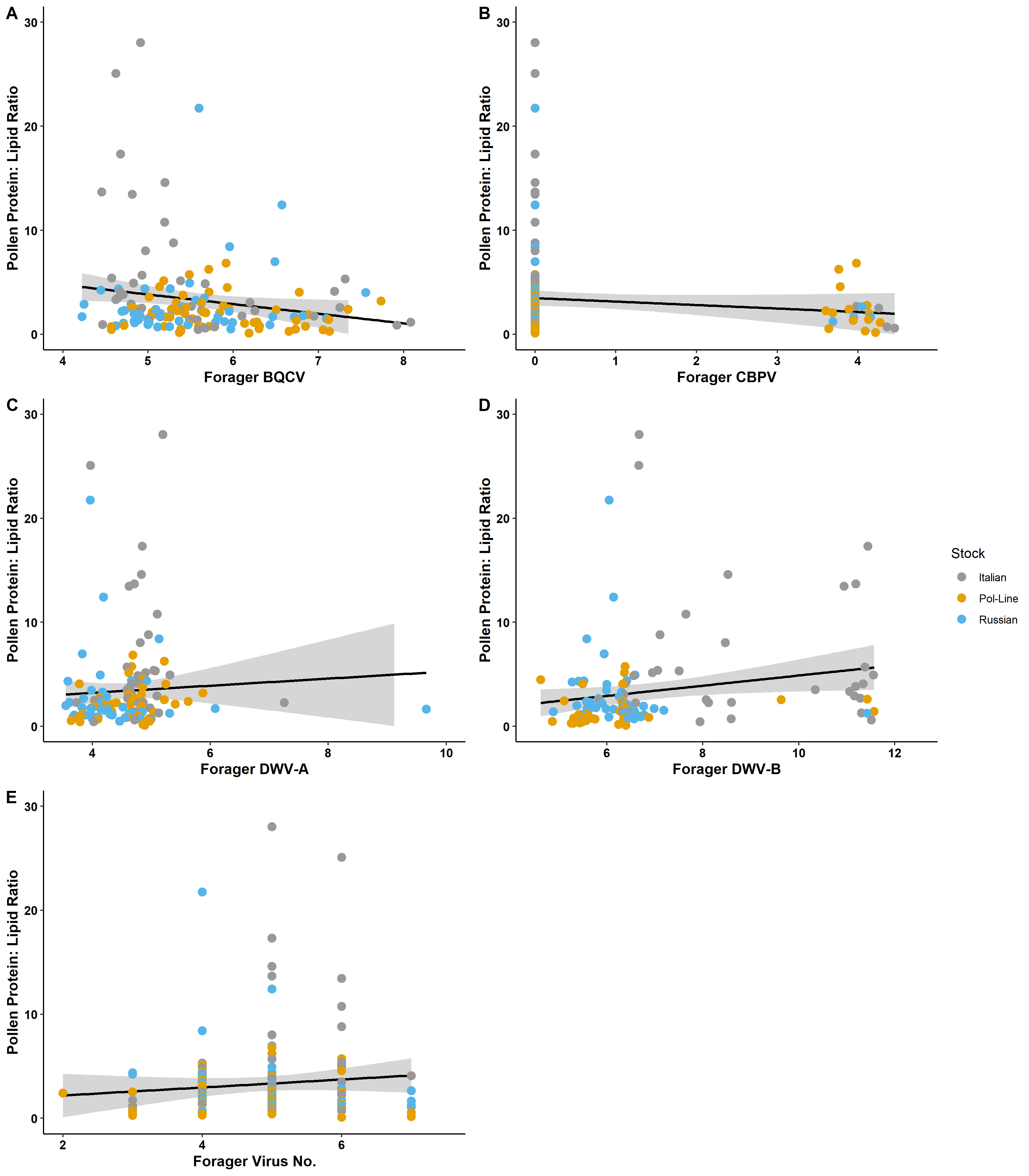


**Figure S2.** Nectar weight (mg) collected by individual foragers relative to log-transformed forager levels of (A) BQCV, (B) CBPV, (C) DWV-A, and (D) DWV-B.


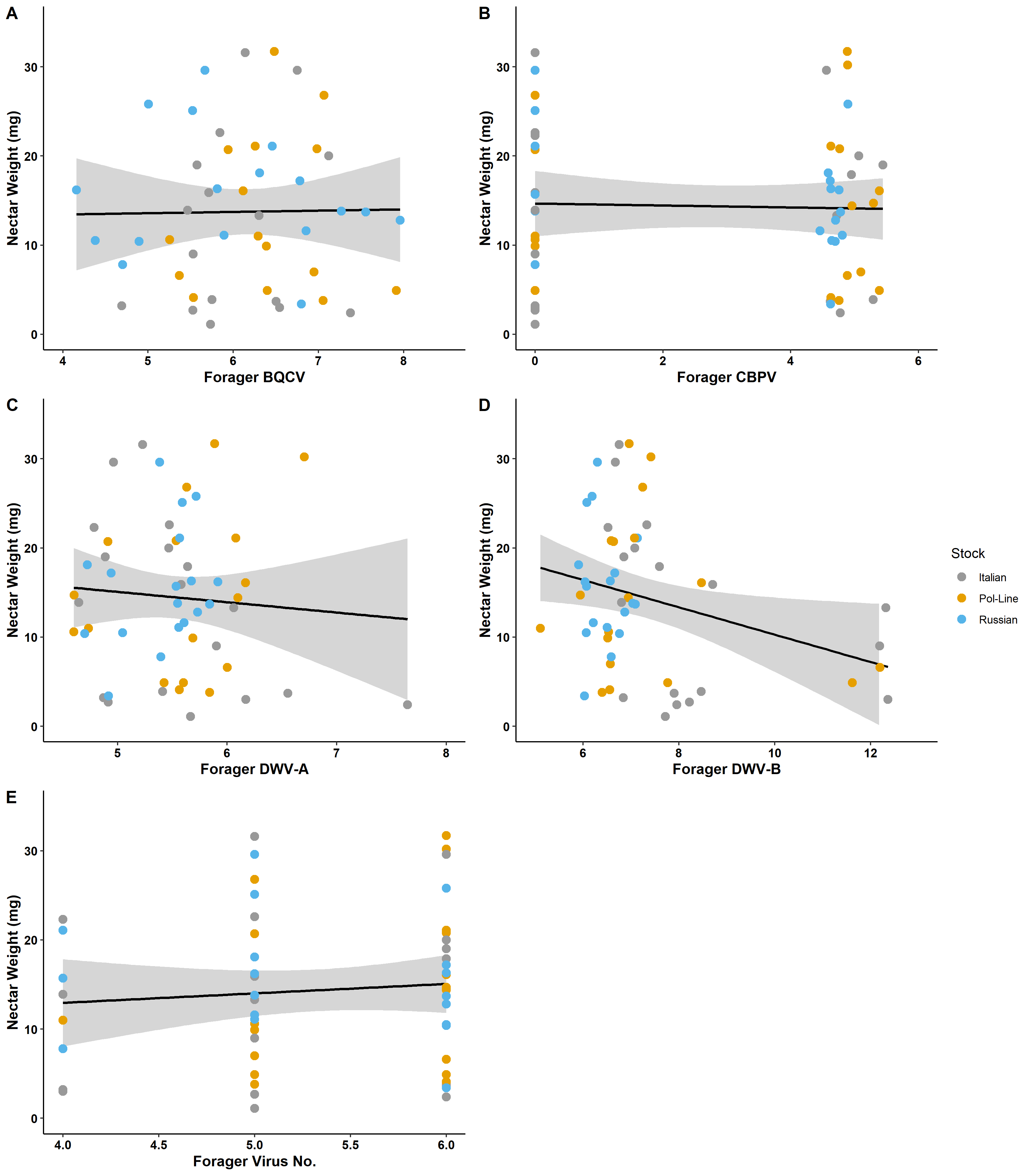


**Figure S3.** Nectar sugar content (Brix) collected by individual foragers relative to log-transformed colony levels of (A) BQCV, (B) CBPV, (C) DWV-A, and (D) DWV-B.


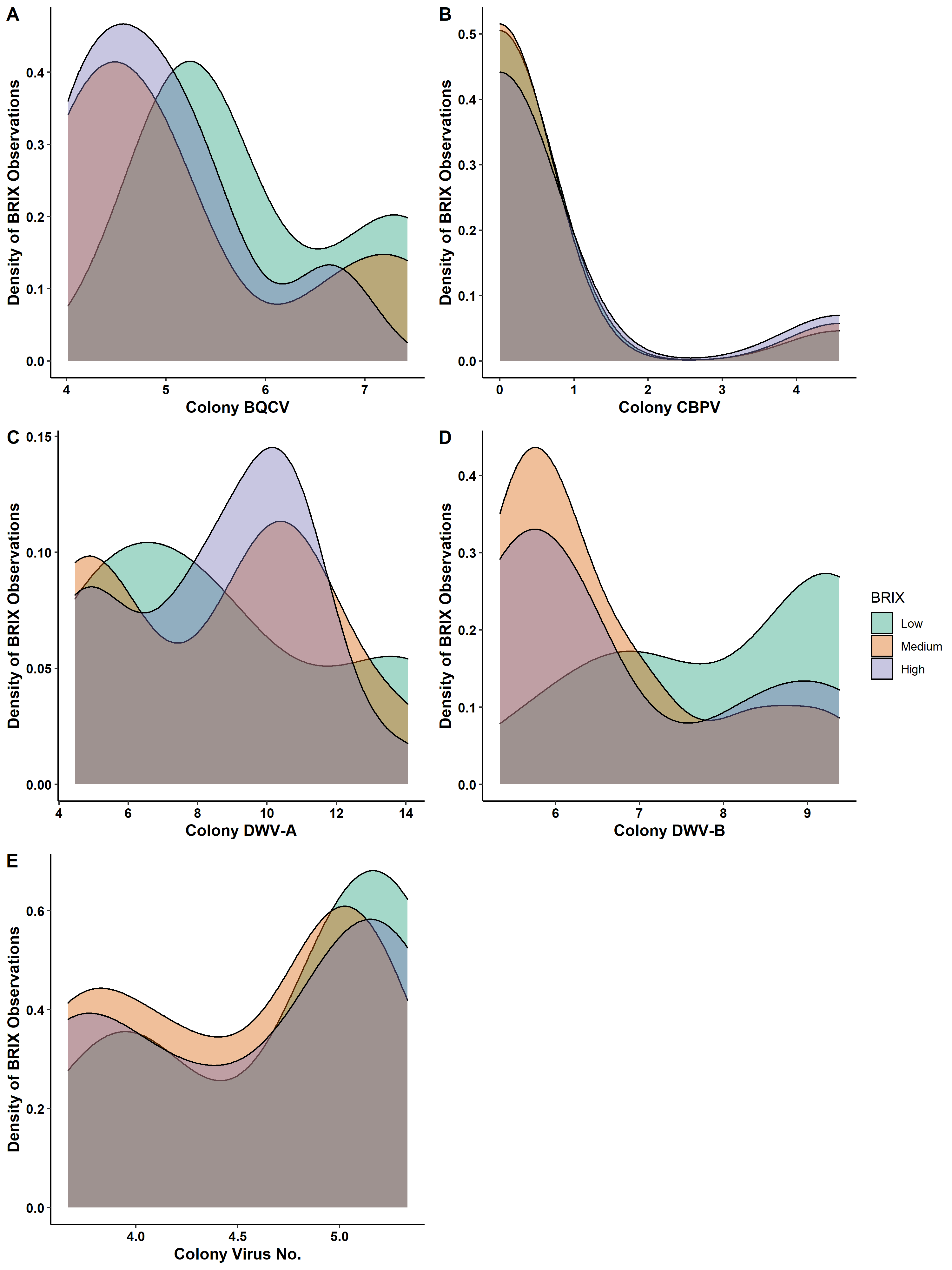


**Figure S4.** Nectar sugar content (Brix) collected by individual foragers relative to log-transformed forager levels of (A) BQCV, (B) CBPV, (C) DWV-A, and (D) DWV-B.


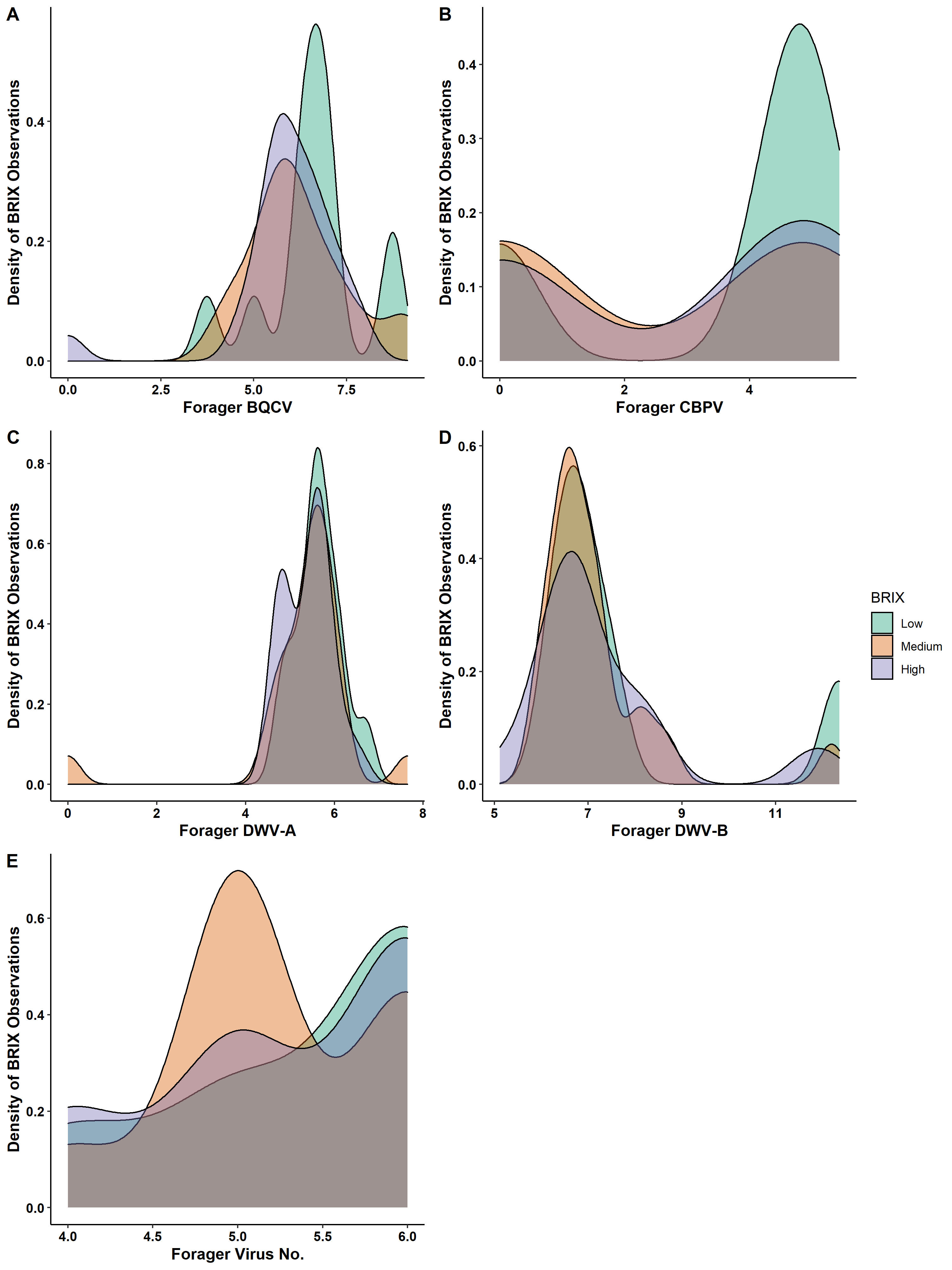


**Figure S5.** P:L ratio of individually collected pollen relative to log-transformed colony DWV-A levels relative to the three genetic stocks of bees tested. Italian bees are considered susceptible to *Varroa* mites while Pol-Line and Russian bees have been bred for mite resistance.


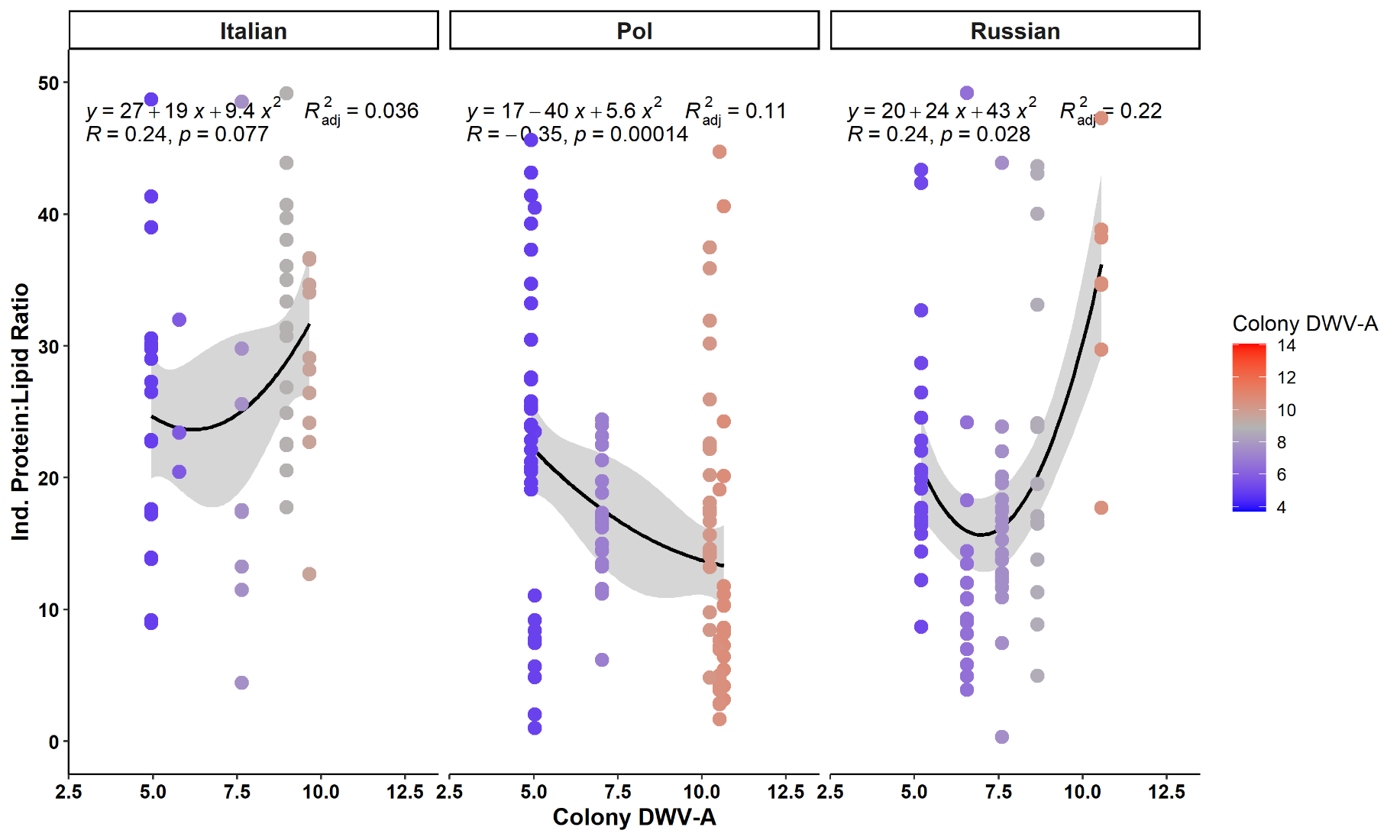


**Figure S6.** P:L ratio of individually collected pollen relative to log-transformed colony DWV-B levels relative to the three genetic stocks of bees tested. Italian bees are considered susceptible to *Varroa* mites while Pol-Line and Russian bees have been bred for mite resistance.


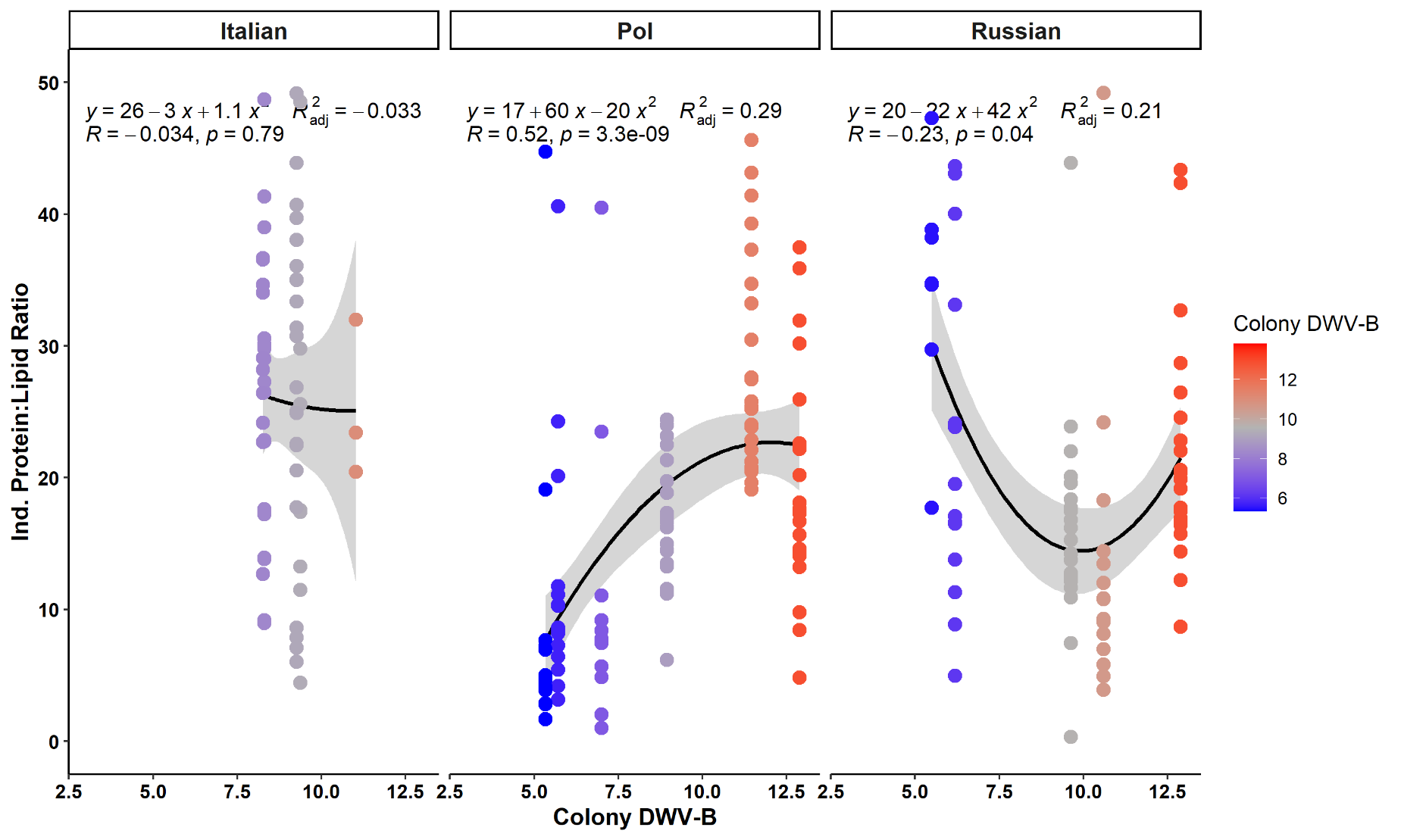


**Figure S7.** Nectar load weight (mg) relative to log-transformed colony BQCV levels relative to the three genetic stocks of bees tested. Italian bees are considered susceptible to *Varroa* mites while Pol-Line and Russian bees have been bred for mite resistance.


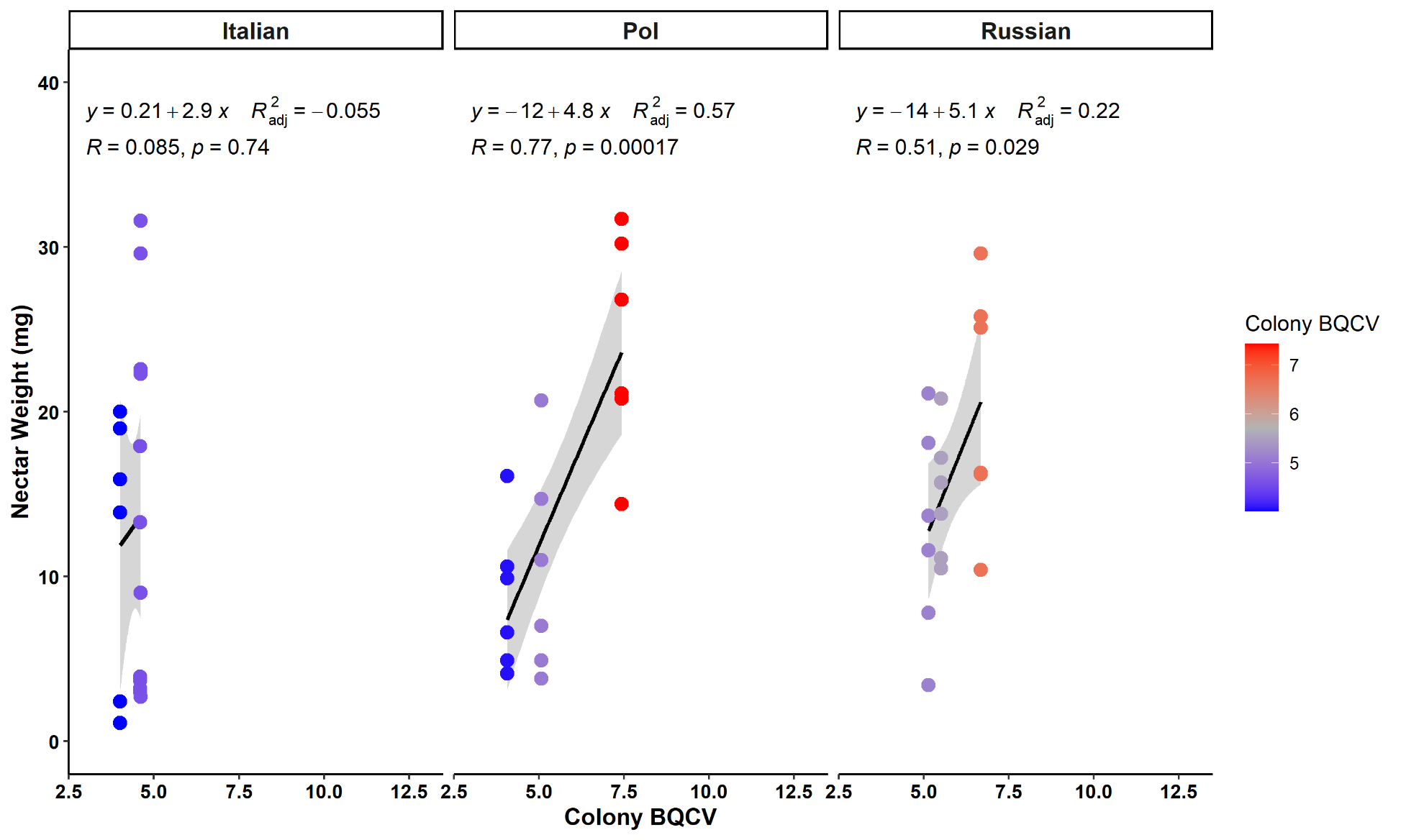
**Figure**

**Figure S8.** Nectar load weight (mg) relative to log-transformed colony DWV-A levels relative to the three genetic stocks of bees tested. Italian bees are considered susceptible to *Varroa* mites while Pol-Line and Russian bees have been bred for mite resistance.


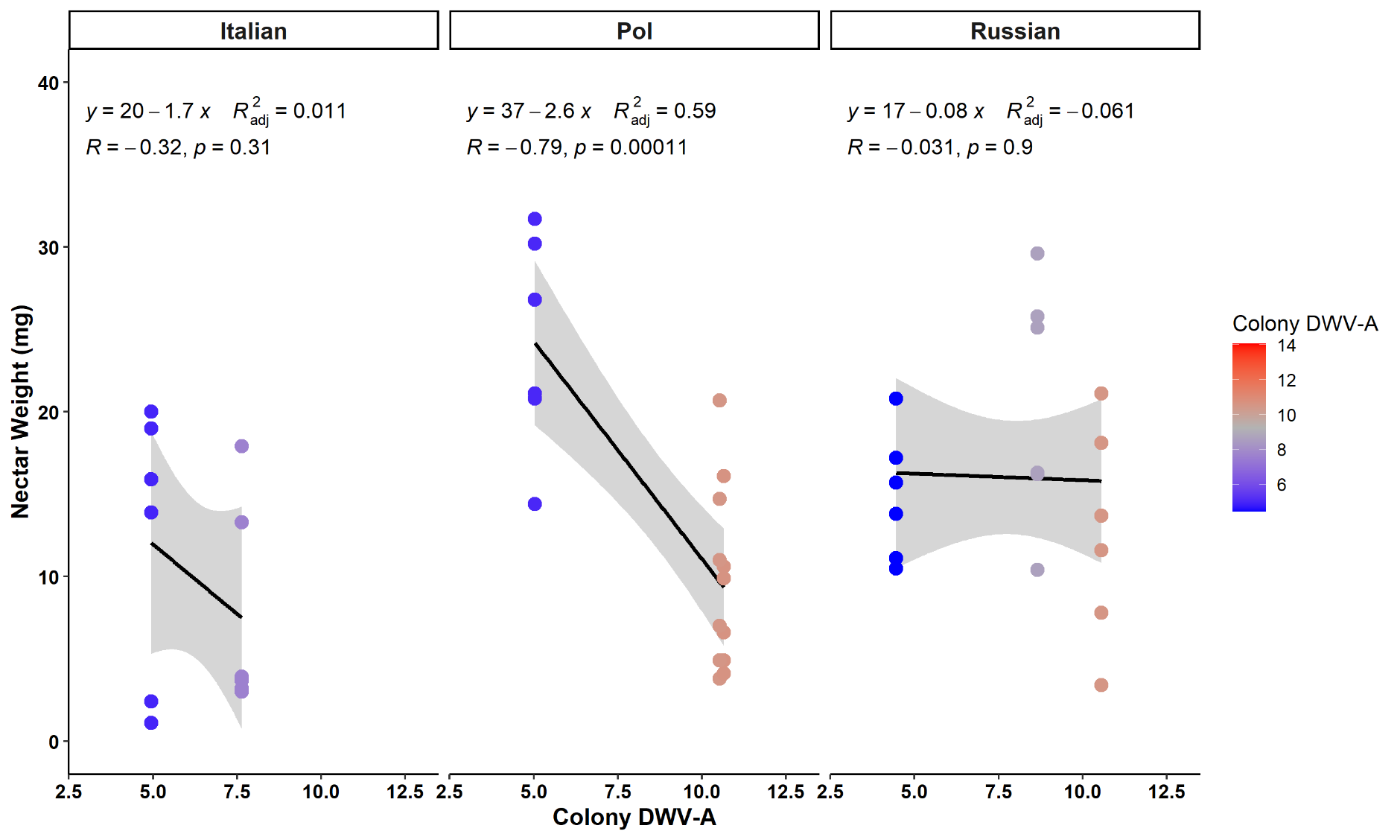


**S9.** Nectar load weight (mg) relative to log-transformed colony DWV-B levels relative to the three genetic stocks of bees tested. Italian bees are considered susceptible to *Varroa* mites while Pol-Line and Russian bees have been bred for mite resistance.


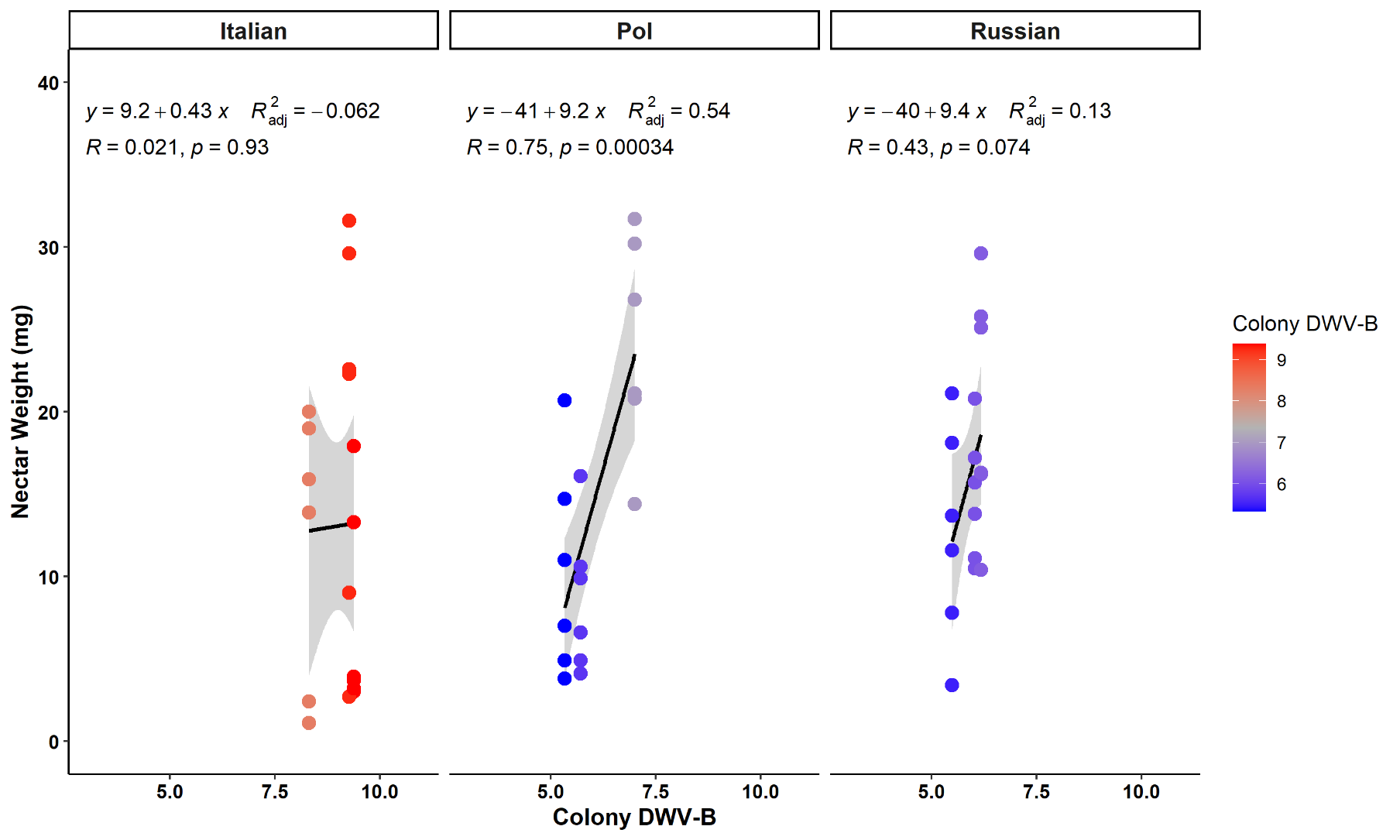

Supplement: Supplementary file 1 [file Data_Sheet_1.docx]
